# Genetic structure of *Spirometra mansoni* (Cestoda: Diphyllobothriidae) populations in China revealed by a Target SSR-seq method

**DOI:** 10.1186/s13071-022-05568-1

**Published:** 2022-12-23

**Authors:** Fang Fang Xu, Wen Qing Chen, Wei Liu, Sha Sha Liu, Yi Xing Wang, Jing Chen, Jing Cui, Xi Zhang

**Affiliations:** 1 Department of Parasitology, School of Basic Medical Sciences, Zhengzhou University, Zhengzhou, 450001 Henan China; 2 Research Center for Parasites and Vectors, College of Veterinary Medicine, Hunan Agricultural University, Changsha, 410128 Hunan China

**Keywords:** Cestode, Plerocercoid, Genotyping, Genetic diversity, SSR-seq

## Abstract

**Background:**

In China, the plerocercoid of the cestode *Spirometra mansoni* is the main causative agent of human and animal sparganosis. However, the population genetic structure of this parasite remains unclear. In this study, we genotyped *S. mansoni* isolates with the aim to improve current knowledge on the evolution and population diversity of this cestode.

**Methods:**

We first screened 34 perfect simple sequence repeats (SSRs) using all available omic data and then constructed target sequencing technology (Target SSR-seq) based on the Illumina NovaSeq platform. Next, a series of STRUCTURE. clustering, principal component, analysis of molecular variance and TreeMix analyses were performed on 362 worm samples isolated from 12 different hosts in 16 geographical populations of China to identify the genetic structure.

**Results:**

A total of 170 alleles were detected. The whole population could be organized and was found to be derived from the admixture of two ancestral clusters. TreeMix analysis hinted that possible gene flow occurred from Guizhou (GZ) to Sichuan (SC), SC to Jaingxi (JX), SC to Hubei (HB), GZ to Yunnan (YN) and GZ to Jiangsu (JS). Both neighbor-joining clustering and principal coordinate analysis showed that isolates from intermediate hosts tend to cluster together, while parasites from definitive hosts revealed greater genetic differences. Generally, a *S. mansoni* population was observed to harbor high genetic diversity, moderate genetic differentiation and a little genetic exchange among geographical populations.

**Conclusions:**

A Target SSR-seq genotyping method was successfully developed, and an in-depth view of genetic diversity and genetic relationship will have important implications for the prevention and control of sparganosis.

**Graphical Abstract:**

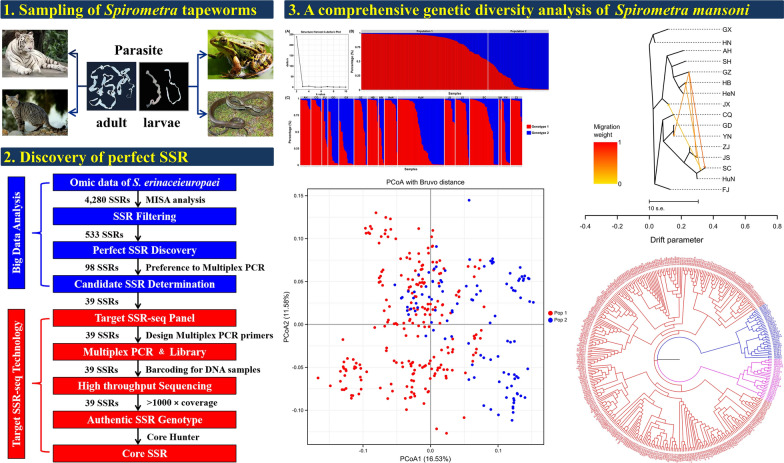

**Supplementary Information:**

The online version contains supplementary material available at 10.1186/s13071-022-05568-1.

## Background

Parasitic tapeworms of humans and animals cause diseases of major socio-economic importance around the world [1]. Several of these remain neglected in terms of research and control, such as *Spirometra mansoni* (Cestoda: Diphyllobothriidae) [2]. The plerocercoid larvae of *S. mansoni* can lead to a serious parasitic zoonosis in humans known as sparganosis by invading the subcutaneous tissues, abdominal cavity, eye and central nervous system, causing local tissue damage, blindness, paralysis, and even death [3]. Human infection results mainly from the ingestion of uncooked frogs or snakes infected with plerocercoids, drinking water contaminated with copepods that have been infected with procercoids or direct contact of frog or snake flesh with an open wound [4]. Most cases of sparganosis have reported in Eastern and Southeastern Asia [5]. On a country basis, China has the largest number of sparganosis cases in the world, with > 1300 reported cases of human sparganosis in 26 out of 34 provinces, autonomous regions and municipal districts [6]. In recent years, the number of local cases have increased in several districts of China, and sparganosis has even been termed as an emerging zoonosis [4, 7, 8].

Knowledge of the extent of intraspecific genetic diversity of *S. mansoni* is not only useful for understanding the dynamics of individual infections but also valuable for the prevention and control of sparganosis. Several pioneer studies have investigated the genetic diversity of *Spirometra* tapeworms. Jeon and Eom [9] performed a genetic variability analysis of *Spirometra* species from Korea, China and Japan using two mitochondrial gene sequences (cytochrome* c* oxidase subunit 1 [*cox1*] and cytochrome b [*cytb*]). The study identified 148 (*cox*1) and 83 (*cytb*) haplotypes within 239 and 213 isolates, indicating that mitochondrial haplotypes of *Spirometra erinaceieuropaei* and *Spirometra decipiens* were found in the three Asian countries [9]. Based on *cox1* sequences, Yamasaki et al. [10] suggested two distinct *Spirometra* species, Type I and Type II, are present in Asia, neither of which is close to the likely European “*S. erinaceieuropaei.*” Kołodziej-Sobocińska et al. [11] found distinct genetic separation of the Polish and Chinese populations of *Spirometra* isolates based on *cox1* and *cytb*, and suggested that isolates from Polish and Chinese populations should be two species. Using a global full-length DNA sequence dataset of *cox1*, Kuchta et al. [3] performed a comprehensive phylogenetic analysis of *Spirometra* tapeworms and suggested that there are at least six distinct lineages of the genus. In China, the genetic diversity of isolates of *Spirometra* tapeworms from different geographical locations and different hosts (frogs and snakes) have been investigated in recent years [12–16]. Although previous studies have provided valuable information that contribute to a better understanding of the genetic diversity of *Spirometra* tapeworms, there is room for improvement: (i) previous studies have relied on relatively small sample sizes with few representative isolates; (ii) the isolates used were collected mainly from intermediate hosts (frogs or snakes), with few samples from final hosts included; (iii) previous estimates of genetic diversity were based on one or only a few traditional mitochondrial markers (mostly *cox*1 and *cyt*b); and (iv) no high-throughput sequencing method was applied. Therefore, our knowledge of the population diversity of *S. mansoni* obtained from different geographical areas of China is still fragmented.

In the study reported here, we explored the genetic diversity of *S. mansoni* using a high-throughput simple sequence repeats (SSRs) genotyping method. SSRs, also known as microsatellites, are highly variable repetitive elements with nucleotide motifs of 1–6 bp and are ubiquitous in most eukaryotic organisms [17]. SSRs have been verified as suitable markers for inferring genetic variance and population differences in cestode species [14, 18–22]. However, traditional analysis based on gel electrophoresis cannot distinguish base differences or changes correctly in SSR amplicons, often causing false positive or false negative results in SSR detection [23]. Recently, an increasing number of studies have validated that genome-wide SSRs, especially perfect SSRs, exhibit stable motifs and conserved corresponding flanking sequences [17, 24–26]. Therefore, the identification of perfect SSRs is critical in microsatellite analysis, such that amplification of the appropriate PCR products can be ensured in genetic research applications. In this study, we used a genotyping method named Target SSR-seq, which combines high-throughput sequencing with genome-wide perfect SSRs that exhibit stable motifs and flanking sequences. Target SSR-seq enables the genotyping of targeted SSR loci simultaneously in a large number of samples with high coverage, using a single Illumina lane [27]. Moreover, by adding sequencing adapters and dual barcode tags, the SSR genotypes were determined directly from the deep sequencing of PCR products [28].

In order to conduct a comprehensive genetic diversity analysis of *S. mansoni* isolates, we collected a total of 368 isolates from 76 different geographical locations in China, which encompassed 16 out of 26 endemic regions of human sparganosis in the country. Specifically, the following objectives were addressed: (i) development of a high-throughput SSR genotyping (Target SSR-seq) method sensitive enough to genotype *S. mansoni*; and (ii) exploration of the genetic structure of *S. mansoni* populations with broad representative samples.

## Methods

### Ethical statement

This study was performed strictly based on the recommendations of the Guide for the Care and Use of Laboratory Animals of the National Health Commission of China. The protocol was approved by the Life Science Ethics Committee of Zhengzhou University (Permission No. 2020-0704).

### Sample collection

**Fig. 1:**
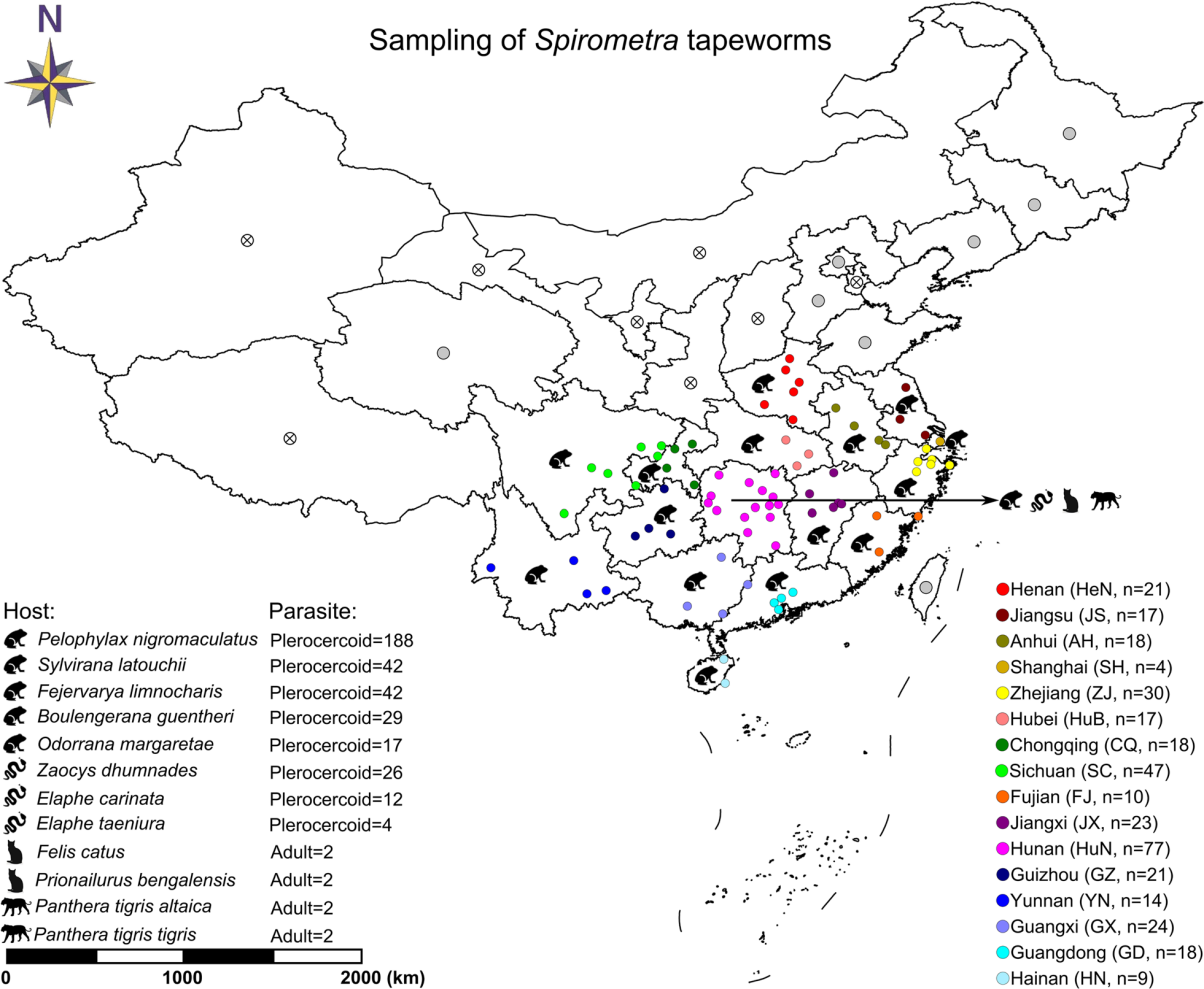
Map of collection localities and host information for *Spirometra mansoni* isolates. The light-gray circles indicate endemic regions of human sparganosis in China where samples were not collected. The circles marked with an 'x' indicate non-endemic regions of human sparganosis in China

Human sparganosis is endemic in 26 provinces/autonomous regions/municipalities in China [5]. From July 2013 to September 2020, we collected *S. mansoni* isolates in 16 of these regions. For the 10 endemic regions where no samples were collected, we surveyed wild frogs for *S. mansoni* infections in Heilongjiang, Jilin, Liaoning, Beijing, Hebei, Shandong and Qinghai (excluding Taiwan, Hong Kong and Macao), but found no parasite-positive frog. In total, 368 samples isolated from 12 different hosts in 76 geographical locations were included in this study (Fig. 1). Among the 368 samples, 318 were plerocercoids collected from frogs (*Pelophylax nigromaculatus*, *Sylvirana latouchii*, *Fejervarya limnocharis*, *Odorrana margaretae* and *Boulengerana guentheri*), 42 were plerocercoids collected from snakes (*Zaocys dhumnades*, *Elaphe carinata* and *Elaphe taeniura*) and eight were adults collected from felids (*Panthera tigris tigris*, *P. tigris altaica, Prionailurus bengalensis* and *Felis catus*). The precise locality, origin and date of collection of each sample are presented in Additional file 1: Table S1. The methods used to collect plerocercoids in frogs and snakes have been described previously [14, 15]. In brief, frogs or snakes were euthanized using ethyl-ether anesthesia and then weighed and skinned. The presence of plerocercoids in muscles and subcutaneous tissues was carefully observed visually. Once identified, the worms were isolated using small scissors and forceps and placed in phosphate-buffered saline to count and observe their shapes and movements. The plerocercoid was identified as *S. mansoni* by molecular genotyping with bidirectional sequencing of the mitochondrial *cox*1 gene (Additional file 1: Table S2) [3, 29]. The definitive hosts (cats and tigers) for collecting adult worms came from a wildlife zoo park in Changsha City, Hunan Province. All animals died of natural causes, and necropsies were carried out under approved protocols by the wildlife zoo park technical office. The intestinal tracts of the analyzed carnivores were carefully removed from each carcass and subsequently isolated by ligatures (pylorus and rectum). Examination of the intestinal content was performed as described by Arrabal et al. [30]. All collected samples were washed extensively in physiological saline, snap-frozen in liquid nitrogen, and then stored at – 80 ℃ for further study.

### Development of perfect SSR markers

**Fig. 2:**
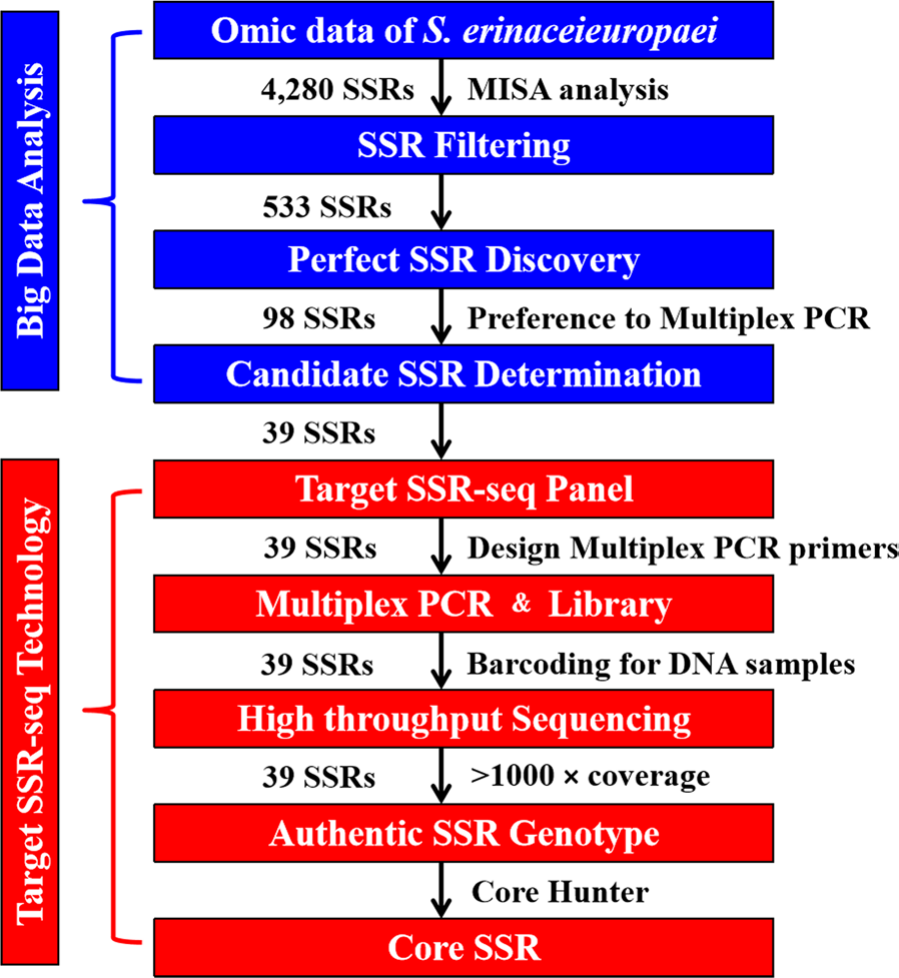
Target SSR-seq pipeline. Schematic workflow of perfect SSR selection, Multiplexed PCR design, high-throughput sequencing, and SSR genotype. MISA, MIcroSAtellite Identification Tool; SSR, simple sequence repeat

A method for SSR genotyping using a target sequencing technology called Target SSR-seq was used to explore the genetic structure of *S. mansoni* populations in China (Fig. 2). The first step was to analyze the transcriptome data (https://www.ncbi.nlm.nih.gov/bioproject/PRJNA761840) [31] to uncover genome-wide SSRs using the MISA-web tool [32]. The search was carried out using the following parameters: (i) > 9 repeat units for mononucleotide microsatellites; (ii) ≥ 4 repeat units for di-, tri-nucleotide microsatellites; (iii) ≥ 3 repeat units for tetra-, penta- and hexa-nucleotide microsatellites; and (iv) repeat length up to 100 bp. The poly-A and poly-T sequences at the terminal regions of the unique transcripts (UTs) were removed before searching. Second, the publicly available expressed sequence tags (ESTs) were searched and retrieved from the NCBI EST database. MISA (MIcroSAtellite Identification Tool) was also used to search for microsatellite loci in the EST sequences. The following parameters were used to identify perfect SSRs (i.e. those that exhibit stable motifs and have conserved corresponding flanking sequences): (i) SSR motif length < 50 bp; (ii) no INDELs, poly regions or SSR loci in the 150-bp flanking sequence; and (iii) even distribution in chromosomes [27]. Primers were designed for SSR loci by Primer3Plus with the following parameters: (i) primer lengths ranging from 18 to 27 bp; (ii) product lengths ranging from 100 to 300 bp; (iii) melting temperature (Tm) ranging from 57 °C to 63 °C; and (iv) GC-content ranging from 40 to 60%, with an optimal value of 50%. Before library construction, we needed to optimize these SSR markers twice. First, each SSR was independently tested and optimized by a single PCR amplification procedure consisting of: 1 cycle of 95 °C for 2 min; 11 cycles of 94 °C for 20 s, 63–58 °C (− 0.5 °C per cycle) for 40 s, 72 °C for 1 min; then 24 cycles of 94 °C for 20 s, 65 °C for 30 s, 72 °C for 1 min; with a final cycle at 72 °C for 2 min. SSR markers which amplified clear bands were selected for further multiplex PCR optimization. According to the multiplex PCR results, the composition and concentration of SSR markers in the panel of multiple PCR were adjusted and optimized to ensure that each SSR marker in the multiplex system could be amplified efficiently and specifically.

### Library construction and high-throughput sequencing

Two rounds of PCR were performed to construct the library. In the first round, the optimized panels were used for multiple PCR amplification using the genomic DNA of each sample as a template. After quality control, the amplified products were mixed, and the quantity of amplified products of each SSR marker was ensured to be equal. In the second round, primers with index sequence were used to add 8 bp of specific label sequences compatible with the Illumina NovaSeq 6000 platform (Illumina Inc., San Diego, CA, USA) to the end of the library. After that, the amplified products of all samples were mixed in equal quantities, and the final FastTarget™ (Genesky Biotechnologies Inc., Shanghai, China) sequencing library was recovered by gumming. The length distribution of fragments in the library was verified using an Agilent 2100 BioAnalyzer (Agilent Technologies, Inc., Santa Clara, CA, USA). After the molar concentration of the library was accurately quantified, the Illumina NovaSeq 6000 platform was finally used for high-throughput sequencing in a 2 × 150-bp double-ended sequencing mode, and each SSR region was sequenced for at least 1000× coverage. In addition, positive and negative controls of PCR amplification were set to assay the repeatability of the Target SSR-seq method. Specifically, in the Target SSR-seq experiment, 3% of the total samples were selected using a double-blind method to set up as positive control, and pure water was used as a negative control. To ensure quality, raw reads must be filtered to get clean reads with FastQC (https://www.bioinformatics.babraham.ac.uk/projects/fastqc/), and subsequent analyses were based on clean reads. The total number of reads was calculated, and the samples were classified. A Perl script “SSRSeq count” str_count.pl (https://github.com/ccoo22/SSRseq_count) was used to process the clean reads to generate an SSR read count table. A Perl script str_type.pl (https://github.com/ccoo22/SSRseq_count) and the SSRSeq V1.1 online SSR genotyping tool (http://bioinfo.geneskybiotech.com/software/ssrseq_type/v1.1/en/) were used to output SSR genotypes.

### Genetic diversity analysis

Genetic information statistics, including observed number of alleles (Na), the effective number of alleles (Ne), Shannon’s information index (I), observed heterozygosity (Ho), and expected heterozygosity (He), were calculated by using the software POPGENE32 [33]. Polymorphic information content (PIC) was calculated by using a Perl script [34]. The formulas are as follows:

$${\text{Ne}} = 1/\sum {P_i^2} ;\ I = - \,\sum {P_i} \,\ln \,{P_i};\,{\text{Ho}}\, = \,\frac{{{N_{{\text{Het}}}}}}{N}\, = \,\frac{{N - {N_{{\text{Hom}}}}}}{N};\,{\text{He}}\, = \,1 - \,\sum {p_i^2} ;$$

$${\text{PIC}}= 1 - \sum {_{i = 1}^l} P_i^2 \cdot - \sum {_{i = 1}^{l - 1}} \sum {_{j = i + 1}^l} 2P_i^2P_j^2\,$$

where *N* is the number of samples,* N*_*Het*_ is the number of homozygous samples,* N*_*Hom*_ is the number of heterozygous samples, *l* is the allele locus and *P*_*i*_ and *P*_*j*_ are the population frequencies of the *i*th and *j*th alleles, respectively.

### Genetic structure analysis

The STRUCTURE v2.3 software program was used to infer the population structure [35]. The population number (*K*) was evaluated from 1 to 10. The optimal *K* value was determined by comparing the LnP (D) and Δ*K* based on the rate of change in LnP (D) [36]. Polysat was used to calculate the genetic distance between pairs of samples and principal coordinate analysis (PCoA) [37]. Nei’s genetic distance was calculated for pairs of subpopulations based on allele frequency. The genetic similarity matrix and genetic distance matrix between subpopulations were obtained. Neighbor-joining (NJ) and ‘ggtree’ in the phangorn package in the R packet (R Foundation for statistical computing, Vienna, Austria) were respectively applied to construct and draw the evolutionary tree [38]. The genetic differentiation coefficient between subpopulations was calculated using the software GenAlEx using allele frequency [39], and population differentiation was measured together with an analysis of molecular variance (AMOVA). The software Treemix was used to infer multiple population splitting and mixing events based on genome-wide allele frequency data [40]. The results were based on the provided materials to draw the maximum likelihood tree of the population and to calibrate migration events in the phylogenetic tree.

### Exploration of core SSR sets for species identification

To screen a minimal number of SSRs for distinguishing the maximal number of *S. mansoni* isolates, we used a script in Perl to calculate the discrimination power for SSRs with the following formula: discrimination power = the number of isolates showing unique genotypes/total isolates. High discrimination power refers to high saturation value and high SSR discernibility. A core set of SSRs with the best discernibility ability was obtained, and the saturation curve was plotted by discrimination power for SSRs. Core Hunter software was used to construct a core collection based on MR (modified Rogers Distance) [41]. MR treats each allele as a separate dimension, which is an improvement on the standard Euclidean distance. The sampling ratios of the software were set as 0.01, 0.02, 0.03, 0.04, 0.05, 0.06, 0.07, 0.08, 0.09, 0.1, 0.2 and 0.3, respectively.

## Results

### Identification of perfect SSRs

A total of 12,481 ESTs related to *S. mansoni* were obtained based on the public EST database, of which 2326 ESTs remained after a redundancy analysis to reduce overestimation. Of these 2326 ESTs, 2059 overlapped and 267 were singletons. Further analysis revealed that the 2059 overlapped ESTs could be clustered into 255 clusters; thus, a total of 522 non-redundant ESTs that contained 915 SSRs were identified after screening the EST database. In addition, combined with 3365 SSRs generated from the transcriptome data, a total of 4280 SSRs were identified by MISA analysis. Of these 4280 SSRs, 1024 (23.93%) were di-nucleotide repeats, 2720 (63.55%) were tri-nucleotide repeats and 101 (2.36%) were tetra-nucleotide repeats (Additional file 1: Table S3). The distribution of SSR motifs revealed that the CT repeat was the most widespread di-nucleotide SSR motif (329/1024), and that the TCT repeat was the most widespread trimer among all tri-nucleotide SSR motifs (217/2760). Based on these identified SSRs, we obtained 98 SSRs after SSR filtering and perfect SSR search. Finally, 39 evenly distributed perfect SSRs were selected to test in Target SSR-seq after primer detection and optimization of the library enrichment system. Detailed information on SSR loci and primers is shown in Table 1.

**Table 1:** Information on simple sequence repeat locui and corresponding primers used in this study

Target SSR locus	Motif	Motif length (bp)	SSR start	SSR end	SSR length (bp)	Motif count	Target length (bp)	Primers
T_01	GCT	3	76	87	12	4	202	F: CTCGCTGCAAAATCTCCAGC
								R: GAATGGGCTGGCCGTTTAAA
T_02	CAC	3	86	97	12	4	205	F: CGATGCTGAGGGCGATTTTC
								R: CGACTCCAGACTCTCTACCGA
T_04	ACT	3	91	108	18	6	217	F: TCCAAAATATTATGCTTTGTCGGCA
								R: GGGCAAACAAGTCCATTTGGT
T_05	GCT	3	67	84	18	6	169	F: GAGATGATTGCGTCCATGGC
								R: AGAAGCCCATGTCATCGTCG
T_08	GCAC	4	30	41	12	3	131	F: CGGCTTGGTACAGCCTTCAA
								R: TCGATGCTGCTCTGATTGCT
T_09	GCAT	4	41	52	12	3	138	F: AGAAATCAGCAGCACCAACA
								R: CGAAAGAGGGGACAGAGCAA
T_11	CAAC	4	68	79	12	3	176	F: GCCCGAATACCGTCGTTTTG
								R: AGTTGGAGCGGATCACCATG
T_12	GTGC	4	77	96	20	5	148	F: GCAAACACAAGGCTTCCCAG
								R: GGTCTCTGGTACGTTTGCCA
T_14	AGA	3	82	93	12	4	168	F: TTGCGCAGACGATAGACAGG
								R: ACGCTGGCTTGTACTCAACA
T_18	GAT	3	46	57	12	4	159	F: CGACCCGGATCCAAACTACC
								R: GGATCGCCCGTATGATTCCC
T_19	TGCA	4	78	89	12	3	241	F: TGCCTCGATTTGGTAAGCACT
								R: CTCTGCTTAAGCGACCGTGA
T_21	GAT	3	55	66	12	4	163	F: GATGCAGGTGACGAGGATGA
								R: GTTTGCTCCCCTCTCCTTCC
T_23	GGA	3	49	60	12	4	171	F: GCTGGAGAATGCCACTCACA
								R: GCTTCCTCATTCGACTCCGT
T_24	ACC	3	80	91	12	4	171	F: ACCGTTACAGACAACTGAACCA
								R: AGCTCAACTCGTGAATCCGG
T_27	CGA	3	54	65	12	4	203	F: GTCTGGGAAGATGGCGTTGA
								R: TCTGTGAGCAAGTGAAGGGC
T_30	GCA	3	86	100	15	5	203	F: ACCGGCATCAACATCAGTCA
								R: GTAGTCCTCGGGGTGTTGTG
T_31	CCT	3	83	94	12	4	185	F: CGTCACCAAATTGAACAGCGT
								R: TTCAGCACCATTTTGCAGGC
T_32	GAG	3	38	55	18	6	166	F: GGAGATGCGATTTCCGGGTT
								R: TCCAACGCAGATGCAACCTA
T_35	CAG	3	95	109	15	5	232	F: CACAGTTCAGGCTGGCAGTA
								R: GGTCTGAGCGTCCATGTGAT
T_36	GAG	3	74	88	15	5	173	F: CACCCCTGGAGGTCTGGATA
								R: GGTCGATTTCGCATTCAGCC
T_38	TTC	3	99	113	15	5	232	F: TTCCCTCCTACAGCGAACCA
								R: GTAGCCTACGACGCCATCTC
T_39	AGC	3	30	41	12	4	157	F: TTTCGCCTCCTGCACCAG
								R: ACTGGCGAGCATGCAGAG
T_40	CGA	3	58	72	15	5	160	F: CGCTAAGCCGGTTGTTGC
								R: CGCCAGGTCTCCGTGAAG
T_41	GCA	3	86	103	18	6	211	F: AAAAGGTGCGTCGTATCCGT
								R: TGCGGCTGACTGTACATCAA
T_42	TGG	3	79	93	15	5	244	F: CCCAACTGTATCCCCATCGG
								R: AGTAAGCTTCTGCGCCATGA
T_43	GAG	3	77	91	15	5	264	F: TACTGGTCTCTCGGGCAAGA
								R: GGTCCGTAGTCGTCGTCATC
T_44	TCC	3	118	138	21	7	215	F: CGCCTCTGGAAGAAGAAGGG
								R: CGTTTGCGCCAGTAACTCTC
T_45	AGAC	4	54	65	12	3	164	F: CGTAAGCGTTGGAGGAAGGT
								R: CGACCAGAGTTCACCTTCCC
T_48	TCC	3	102	113	12	4	209	F: TCCACGCCAAGGAGAATGAC
								R: CAGGGGCTTGTAGTGACCG
T_49	GGA	3	113	124	12	4	205	F: CGTCCGACAGTTGGCAGAAA
								R: CCACACGCGTTTTCACAGTC
T_55	GAAA	4	61	72	12	3	213	F: TGTGACATAATGGTCCGTGTCA
								R: CCGACCGTTAAGCTGGACAA
T_57	ACG	3	53	70	18	6	209	F: TGATATGCGAGCAACGGTGT
								R: GGCGAGGAAGTTGACACGAT
T_59	ACG	3	90	101	12	4	209	F: CGGTCATCTTCCTGCATCGA
								R: TGTGTGATCAGCGACCAGTG
T_60	CTT	3	75	86	12	4	208	F: GTCCTCAGCACCTGGAACAT
								R: GCCACTCAAGGGTAGGTTGT
T_61	GAT	3	84	95	12	4	233	F: CGCAAGCCGTTCAGTACAAA
								R: CAGACCCAGACCGCAGAC
T_62	ATG	3	83	97	15	5	173	F: GGTGAGAGGGCTGAAACACA
								R: CGCTCTACAAGGGTCTCGTC
T_63	CTC	3	87	107	21	7	173	F: AGCTGTACAATTGCCGTGGA
								R: TCGACTGCCATCCCTCAAAC
T_64	GCA	3	106	117	12	4	167	F: CAAAGCCCTGTCTCCGGAG
								R: GGGGACTGGAGAGGCAGA
T_65	CCA	3	77	91	15	5	207	F: CTGCGACAGTTGCCTCCTTA
								R: CGGAAACTCAGTGTGTGGCA

*F* Forward,* R* reverse,* SSR* simple sequence repeat

### Genotyping analysis of SSR-seq

**Fig. 3:**
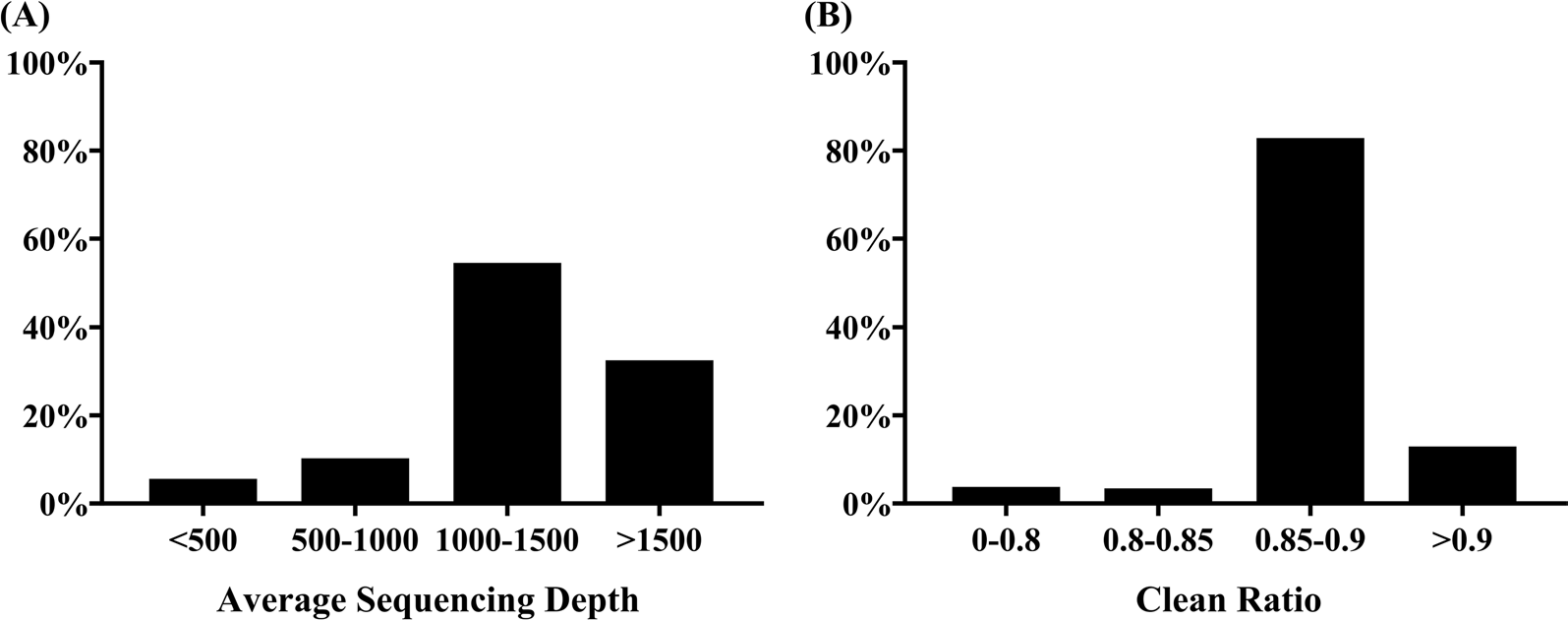
Results of Target SSR-seq genotyping analysis. The distribution of average sequencing depth (**a**) and clean ratio (**b**) for 362 samples

During the high-throughput sequencing of 368 *S. mansoni* isolates, six samples failed due to the low quality of the specimens. The average sequencing depth per SSR capture in 309 samples (85.4%) was > 1000× (Fig. 3a). The clean ratio was > 0.8 for almost all of the samples (97%) (Fig. 3b), indicating the high quality and accuracy of the sequencing results. Moreover, four of the 39 SSRs exhibited high miss rates (> 20%), probably due to unstable flanking sequences. One SSR was observed as monomorphism in 362 varieties. Ultimately, a total of 34 polymorphic SSRs were obtained for the genotyping of 362 *S. mansoni* varieties.

### Genetic diversity of* S. mansoni* populations

Target SSR-seq captured 170 alleles of 34 target SSR loci in 362 samples. Two motif types, trinucleotide and tetranucleotide, accounted for 88.2% and 11.8% of the 170 alleles, respectively. The number of SSR motif repeats ranged from three to seven, and 55 alleles (32.4%) contained four repeat units. The number of alleles per SSR locus varied from two to 18, with an average of five; however, the average effective number of alleles (Ne) was 2.4763, indicating a non-uniform distribution of alleles in the population (Table 2). The Shannon’s information index (I) varied from 0.1064 to 2.5105, with an average of 0.9455. The observed heterozygosity Ho ranged from 0.0417 to 0.9611, with a mean of 0.527, and most of the SSRs (25) exhibited higher Ho (> 0.4), indicating a high level of genetic variability in 362 *S. mansoni* isolates. Furthermore, the genetic diversity estimated by expected heterozygosity *(*He) varied from 0.0409 to 0.9064 (mean = 0.5004), while the PIC value ranged from 0.0401 to 0.8971 (mean = 0.4508). Overall, the 34 target SSR loci showed various alleles and high polymorphism rates, which were proven to be suitable for the identification of the varieties of *S. mansoni* isolates.

**Table 2:** Characteristics of 34 single sequence repeat markers

Target SSR locus	Sample size^a^	Na	Ne	I	Ho	He	PIC
T_01	722	5	2.4967	1.0691	0.7562	0.6003	0.524
T_02	716	8	4.3066	1.5878	0.8939	0.7689	0.731
T_04	570	18	10.5098	2.5105	0.4842	0.9064	0.8971
T_05	432	4	1.7408	0.7223	0.4028	0.4265	0.3641
T_08	712	3	2.6697	1.0317	0.5955	0.6263	0.5481
T_09	724	4	1.9464	0.9185	0.4475	0.4869	0.447
T_11	720	3	2.1004	0.8666	0.5917	0.5246	0.4479
T_12	724	4	1.9567	0.9311	0.5939	0.4896	0.4506
T_14	724	2	1.0539	0.1214	0.0525	0.0512	0.0498
T_21	722	4	1.3479	0.5136	0.2825	0.2584	0.2411
T_23	708	4	2.6312	1.0288	0.4944	0.6208	0.5416
T_24	710	5	3.2449	1.3492	0.8732	0.6928	0.6434
T_27	718	4	2.095	0.9978	0.6657	0.5234	0.4841
T_30	714	4	1.7712	0.8249	0.4454	0.436	0.4007
T_31	724	6	1.5751	0.7245	0.4116	0.3656	0.3385
T_32	556	5	2.1177	1.0213	0.2122	0.5287	0.4859
T_35	666	6	2.7312	1.1429	0.8679	0.6348	0.5654
T_36	720	6	3.2634	1.2547	0.9611	0.6945	0.6327
T_39	718	8	2.4265	1.1279	0.6964	0.5887	0.5398
T_40	552	5	2.6982	1.14	0.4022	0.6305	0.5645
T_41	724	9	3.6986	1.5405	0.8536	0.7306	0.691
T_42	724	4	1.1146	0.2409	0.1022	0.103	0.0992
T_43	720	3	1.1606	0.3039	0.1472	0.1386	0.1325
T_45	688	3	1.9875	0.7375	0.2936	0.4976	0.3857
T_49	720	3	1.9485	0.688	0.7167	0.4875	0.3697
T_55	720	3	1.0426	0.1064	0.0417	0.0409	0.0401
T_57	718	5	2.3392	1.0897	0.4652	0.5733	0.5217
T_59	724	7	4.5298	1.6179	0.6685	0.7803	0.744
T_60	652	4	1.4452	0.6241	0.3252	0.3085	0.2895
T_61	720	3	2.2046	0.8727	0.8889	0.5472	0.4508
T_62	720	5	2.0961	0.8558	0.55	0.5236	0.4264
T_63	722	5	2.5354	1.0769	0.8504	0.6064	0.5423
T_64	688	4	1.5696	0.711	0.3372	0.3634	0.3341
T_65	720	4	1.8385	0.7974	0.5472	0.4567	0.4043
Mean	692	5	2.4763	0.9455	0.527	0.5004	0.4508

*Na* Observed number of alleles, *Ne* effective number of alleles, *I* Shannon’s information index, *Ho* observed heterozygosity, *He* expected heterozygosity, *PIC* polymorphism information content

^a^The total number of alleles on this locus in all samples

**Fig. 4:**
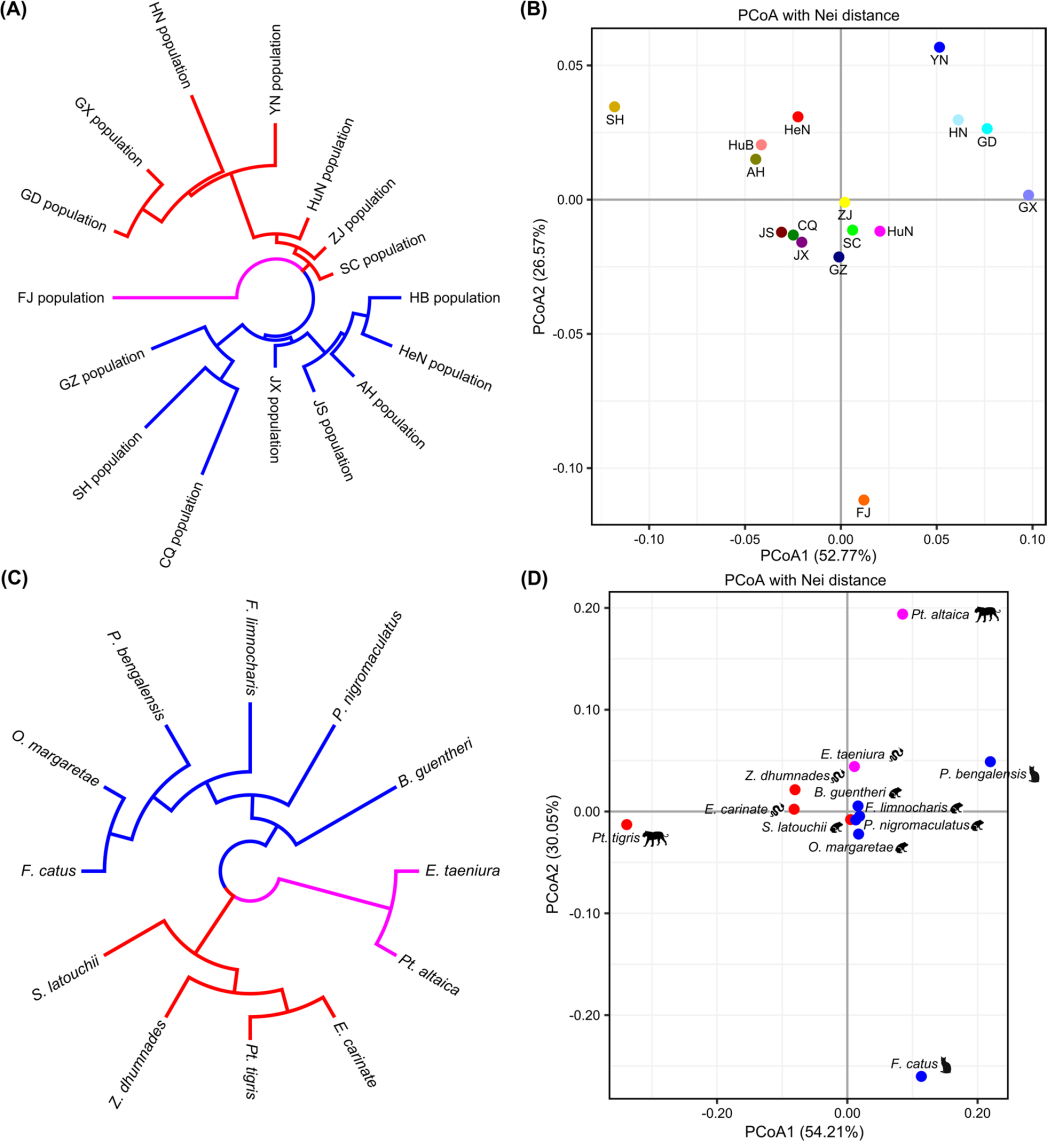
Genetic diversity analysis of isolates from 16 geographical populations and 12 different hosts. **a** Unrooted neighbor-joining tree (NJ) of isolates from 16 geographical populations. The 16 geographical populations (and abbreviations) are the same as in Fig. 1. **b** Principal coordinate analysis (PCoA) describing the relationships of isolates from 16 geographical populations. **c** Unrooted NJ tree of isolates from 12 different hosts. The 12 different hosts (and symbols) are the same as in Fig. 1. **d** PCoA describing the relationships of isolates from 12 different hosts

**Fig. 5:**
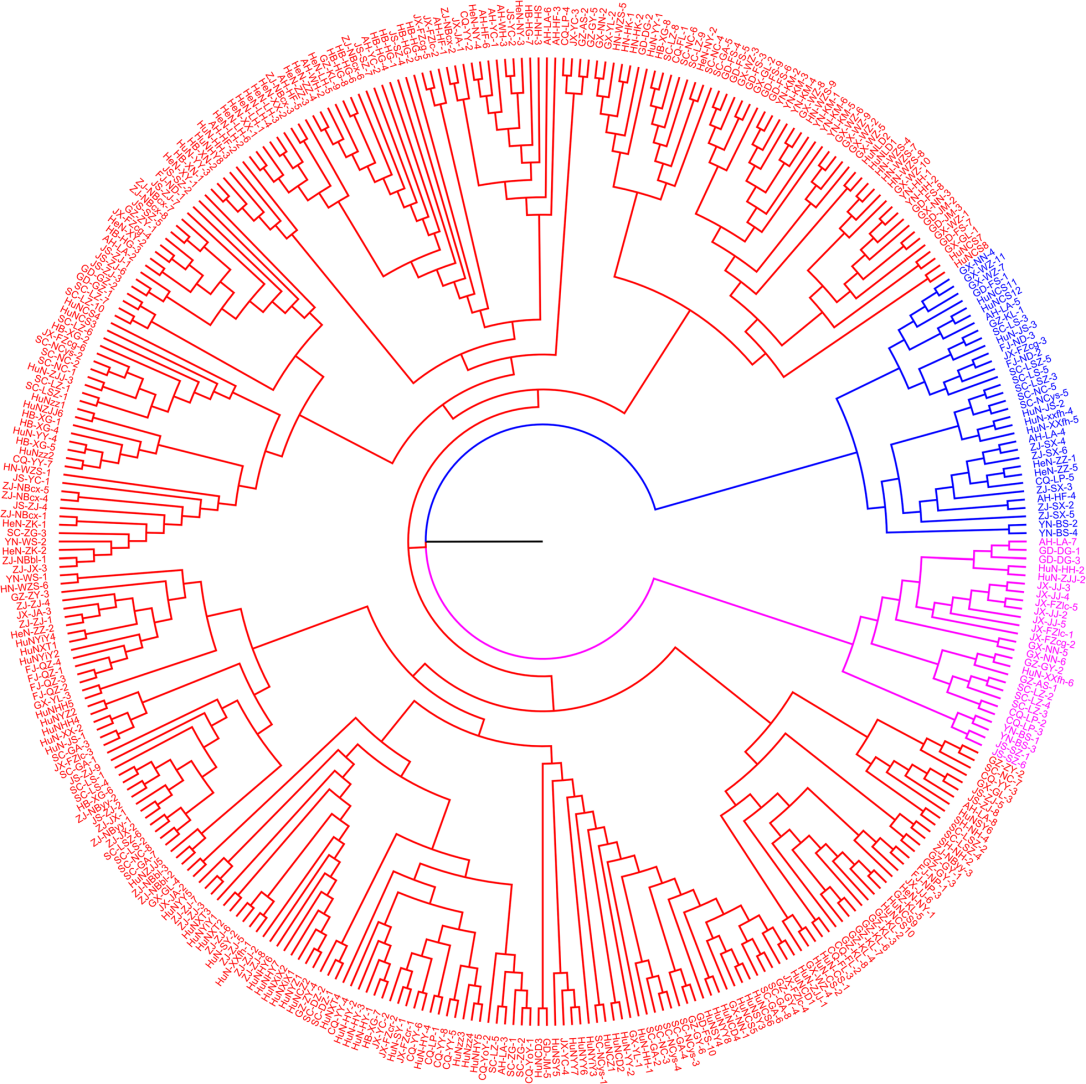
Unrooted NJ tree of 362 *Spirometra mansoni* isolates. Red, blue and pink branches indicate cluster 1, cluster 2 and cluster 3, respectively

For the genetic diversity analysis, we first explored the genetic differences among the 16 geographical populations. The NJ tree based on Nei’s unbiased genetic distance demonstrated that the 16 geographical populations were clustered into three genetic groups (Fig. 4a). The first group mainly included populations from central (Hubei and Henan provinces) and eastern (Anhui, Jiangsu, Jiangxi, and Shanghai) China, with the exception of the Chongqing and Guizhou populations. The second group contained three southwestern populations (Guangxi, Yunnan and Sichuan), three southern populations (Guangdong, Hainan and Hunan) and one population from eastern China (Zhejiang). The third group only consisted of the Fujian (FJ) population from southeastern China. PCoA showed a similar clustering pattern to that of phylogenetic inference (Fig. 4b). Within the PCoA plot, the first principal coordinate accounted for 52.77% of the total variation, while the second principal coordinate accounted for 26.57%. Populations from geographically close regions showed similar distributions in the PCoA plot. However, the FJ population is located far away from the other populations, suggesting larger genetic variation within the FJ population. Next, genetic differences of tapeworms isolated from different hosts were explored (Fig. 4c). Clustering analysis showed that the samples from different hosts were clustered into three genetic groups. The first group included isolates from four frogs (*P. nigromaculatus*, *F. limnocharis*, *O. margaretae* and *B. guentheri*) and two cats (*F. catus* and *P. bengalensis*). The second group contained isolates from one frog (*S. latouchii*), two snakes (*Z. dhumnades* and *E. carinata*) and one tiger (*P.t. tigris*). The third group included isolates from one snake (*E. taeniura*) and one tiger (*P.t. altaica*). The PCoA analysis showed 54.21% and 30.5% explained variance of PCoA1 and PCoA2, respectively (Fig. 4d). The locations of the definitive hosts (cats and tigers) were scattered and far from each other, while the intermediate hosts (frogs and snakes) were concentrated in the middle of the coordinate axis and close to each other, indicating greater genetic variety among the definitive hosts and less genetic variety among intermediate hosts. Moreover, in order to investigate the detailed genetic diversity of each isolate, we performed a phylogenetic analysis using all 362 samples. The unrooted NJ tree revealed three main clusters (Fig. 5). Cluster 1 was the main clade and included 303 samples; cluster 2 and cluster 3 included 33 and 26 samples, respectively.

### Genetic structure of* S. mansoni* in China

**Fig. 6:**
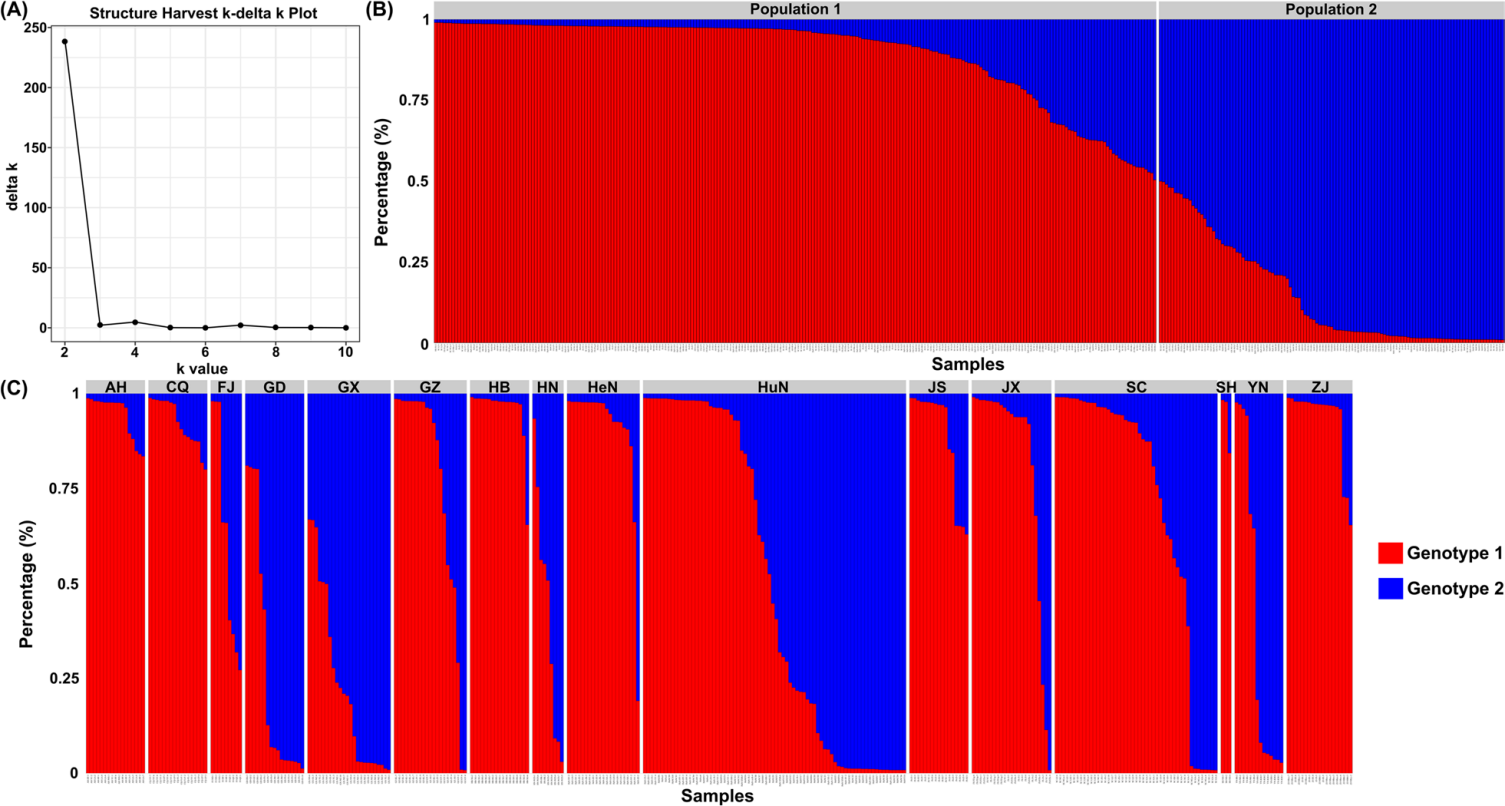
Estimated genetic structure of *S. mansoni* in China as inferred by the STRUCTURE software program on the basis of the data collected on 34 SSR markers obtained from 362 individuals collected from 16 geographical populations (abbreviations as in Fig. 1). **a** Plot of the delta *K* values (population number) generated by the STRUCTURE software program. **b** STRUCTURE plots of 362 individuals grouped by the Q-matrix (estimated membership coefficient for each sample) at *K* = 2. Each isolate is represented by a vertical line, partitioned into the colored segments representing the tapeworm estimated membership fractions in *K*. The same color indicates that the isolates belong to the same group. Different colors for the same isolate indicate the percentage of the genotype shared with each group. **c** STRUCTURE plots of individuals according to 16 geographical populations

**Fig. 7:**
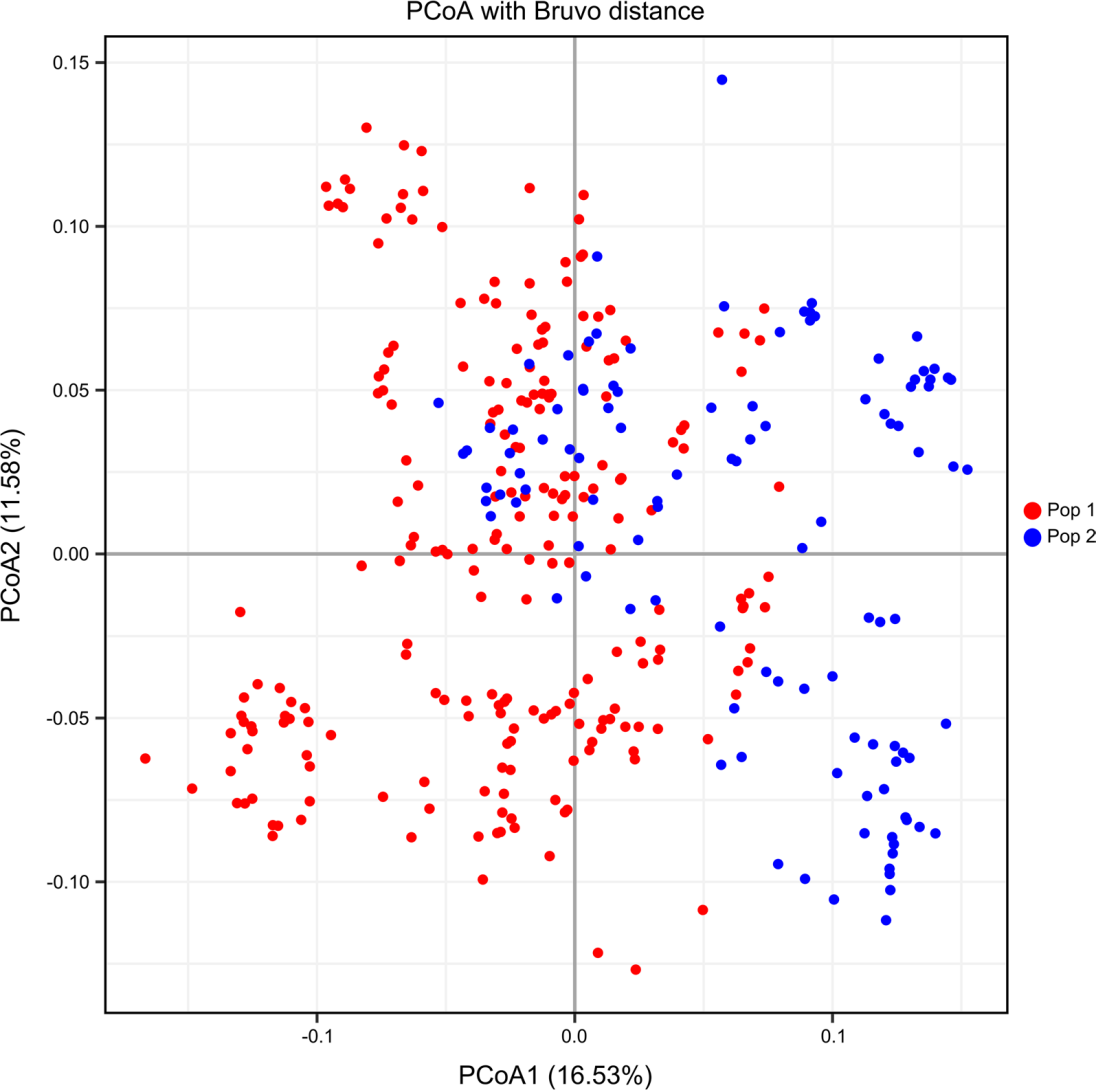
PCoA describing the relationships of 362 *S. mansoni* isolates

The STRUCTURE software program and Evanno’s correction results indicated that 362 isolates were divided into two main populations (Pop1 and Pop2) based on the optimal number of *K* = 2 (Fig. 6a). In general, 245 samples (67.7%) were assigned to Pop1, and the remaining 117 samples (32.3%) were assigned to Pop2 (Fig. 6b). Samples in Pop1 isolated from AH (*n* = 17), CQ (17), FJ (5), GD (5), GX (3), GZ (17), HB (17), HeN (20), HN (4), HuN (37), JS (17), JX (19), SC (36), SH (3), YN (6) and ZJ (22), and samples in Pop2 were collected from FJ (*n* = 4), GD (12), GX (21), GZ (4), HeN (1), HN (5), HuN (39), JX (4), SC (11), YN (8) and and ZJ (8) (see Fig. 1 for abbreviations of geographical locations). Clearly, the STRUCTURE analysis did not divide the 16 populations according to their geographical origin. In contrast, each population showed a mixed membership to the inferred clusters. Isolates from AH (*n* = 17), CQ (17), HB (17), JS (17) and SH (3) revealed one prominent genotype, whereas the remaining isolates indicated the admixture of two genotypes (Fig. 6c). Moreover, the PCoA analysis of 362 samples showed a similar clustering pattern to the STRUCTURE analysis (Fig. 7). All isolates are generally clustered into two main groups (Pop1 and Pop2) with partial overlap among several samples.

### Population differentiation of S. mansoni in China

**Fig. 8:**
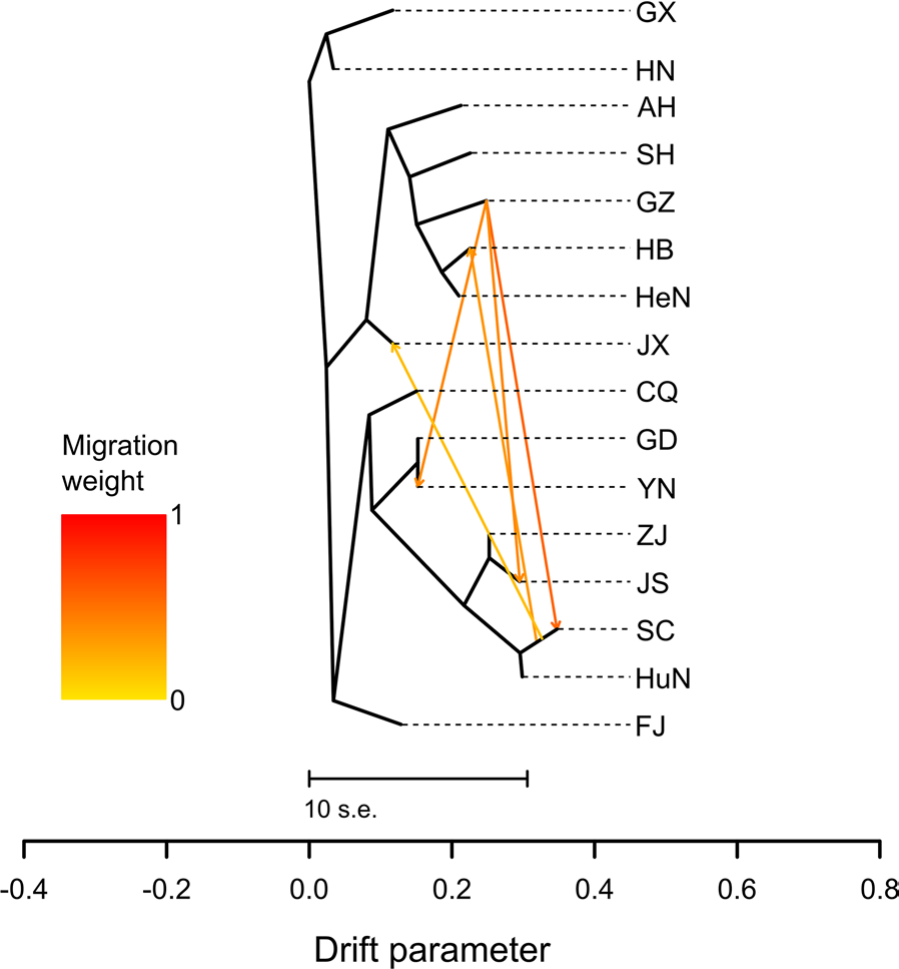
Maximum likelihood tree among 16 geographical populations (See Fig. 1 for abbreviations) constructed with TreeMix. Arrows indicate migration events and are colored according to their migration weight. Horizontal branch lengths are proportional to the amount of genetic drift estimated among populations. The scale bar indicates tenfold the average standard error (*s.e.*) of the values in the covariance matrix

The AMOVA analysis of 34 SSR genotypes in 362 samples indicated that the maximum variation of 94.40621% resulted from differences within populations, while the remaining variation came from “among groups” and “among populations within groups,” accounting for 2.13143% and 3.46236% of the total variance, respectively (Table 3). The pairwise fixation index (*F*_st_) values between specified regions were estimated to measure the population differentiation. Except for *F*_st_ values between several geographical populations (such as ZJ and AH, ZJ and CQ, ZJ and GZ, SC and GZ, ZJ and HuN, SC and HuN), most were within the range of 0.05–0.15, indicating moderate genetic differentiation (Table 4; Additional file 1: Fig. S1). To infer migration events between populations, a maximum likelihood tree was built using TreeMix (Fig. 8). Consistently, the tree topology indicated that all of the geographical populations clustered into three main branches. A large number of migration events were identified among these *S. mansoni* populations. One migration that was detected began from the GZ population to the SC population with a strong migratory signal (migration weight: 0.6). In addition, four migrations with weak signals were detected, including one event that began from SC to JX (migration weight: 0.25), one that began from SC to HB (migration weight: 0.3), one that began from GZ to YN (migration weight: 0.5) and one that began from GZ to JS (migration weight: 0.45).

**Table 3:** Analysis of molecular variance of the *Spirometra mansoni*populations

Source of variation	*df*	Sum of squares	Variance components	Percentage of variation
Among groups	2	101.498	0.17942	2.13143
Among populations within groups	13	264.813	0.29146	3.46236
Within populations	346	5496.774	7.94695	94.40621
Total	361	5863.084	8.41783	100

*df* degrees of freedom

**Table 4:** Estimates of the pairwise fixation index *F*_st_ values between *Spirometra erinaceieuropaei* populations

Population^a^	Population^a^															
	AH	CQ	FJ	GD	GX	GZ	HB	HN	HeN	HuN	JS	JX	SC	SH	YN	ZJ
AH	0.000															
CQ	0.063	0.000														
FJ	0.091	0.083	0.000													
GD	0.083	0.089	0.111	0.000												
GX	0.089	0.089	0.098	0.043	0.000											
GZ	0.064	0.067	0.074	0.076	0.078	0.000										
HB	0.055	0.065	0.098	0.091	0.098	0.069	0.000									
HN	0.109	0.111	0.133	0.082	0.079	0.102	0.108	0.000								
HeN	0.053	0.080	0.108	0.090	0.094	0.070	0.048	0.114	0.000							
HuN	0.067	0.057	0.071	0.058	0.059	0.054	0.069	0.088	0.070	0.000						
JS	0.050	0.064	0.078	0.084	0.089	0.059	0.055	0.110	0.055	0.063	0.000					
JX	0.054	0.053	0.075	0.078	0.082	0.053	0.064	0.108	0.068	0.051	0.051	0.000				
SC	0.060	0.054	0.067	0.061	0.064	0.047	0.059	0.089	0.062	0.037	0.053	0.049	0.000			
SH	0.060	0.073	0.111	0.111	0.113	0.086	0.084	0.131	0.093	0.082	0.079	0.070	0.078	0.000		
YN	0.082	0.083	0.116	0.052	0.059	0.077	0.077	0.084	0.081	0.062	0.082	0.080	0.064	0.101	0.000	
ZJ	0.046	0.043	0.066	0.059	0.059	0.045	0.058	0.089	0.048	0.036	0.042	0.042	0.035	0.064	0.055	0.000

^a^See Fig. 1 for abbreviations of geographical locations

### Core SSR set identification and core samples analysis

**Fig. 9:**
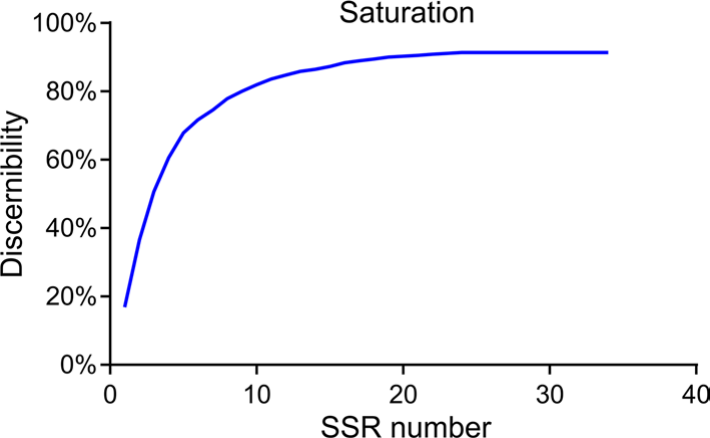
The saturation curve of all SSRs identified in 362 *S. mansoni* isolates. A total of 23 SSRs identified 91.1% of *S. mansoni* varieties

At the end of the study, we intended to explore a core SSR set that will be more suitable and precise for use in the identification of *Spirometra* isolates through further screening of all identified 34 polymorphic SSRs. As a result, a set of 23 core SSRs that could distinguish 91.1% of 362 *S. mansoni* varieties was identified (Fig. 9; Additional file 1: Table S4). Consistent with the above finding, structure analysis based on 23 core SSRs classified the 362 isolates into two populations (Additional file 1: Fig. S2). The PCoA analysis also distinguished the two populations, with PCoA1 explaining 15.37% and PCoA2 explaining 13.39%, respectively (Additional file 1: Fig. S3). The AMOVA analysis showed that the maximum variation mainly came from within populations. (Additional file 1: Table S5). The pairwise *F*_st_ between Pop1 and Pop2 was 0.052. Therefore, the set of 23 core SSRs was sufficient in representing the genetic diversity and identifying Chinese *S. mansoni* varieties.

In addition, considering the huge geographical areas of China, it is hard to sample enough isolates to represent the whole genetic background of Chinese *S. mansoni* populations. Therefore, we want to screen core sample sets in each geographical population based on our collected isolates so that these core samples can represent core or backbone varieties of each geographical population with minimum number of samples. Finally, a total of 76 strains were selected as the core samples of Chinese *S. mansoni* under the sampling ratio of 0.2 and MR of 0.43 (Additional file 1: Table S6).

## Discussion

Simple sequence repeats exist extensively throughout eukaryotic genomes and are widely used for estimating genetic diversity and spatial structure of populations [1]. Traditional microsatellite analysis based on gel electrophoresis usually cannot distinguish base differences or changes correctly in SSR amplicons [23]. In addition, the experimental process of the traditional SSR genotyping method is extremely complicated and time-consuming. Therefore, the research community needs the development of novel methods for SSR discovery and genotyping that require less effort while delivering high accuracy and efficiency. To our knowlegde, our study is the first to use Target SSR-seq in genetic research on medical tapeworm populations. In detail, most of the selected *Spirometra* samples (85.4%) had a > 1000× average sequencing depth, indicating high sequencing depth in this population genetics study. Only six of the 368 samples failed to be genotyped due to the quality of the samples, suggesting high genotyping efficiency. Additionally, the number of PCR cycles in this study was 11, while traditional genotyping methods usually reach up as high as 40 cycles; taking into account that the higher the number of PCR cycles, the higher the possibility of incurring DNA polymerase slippage, the low number of PCR cycles in our study indicate that the PCR quality was highly reliable. Moreover, the Target SSR-seq method processed our multi-target SSR loci (34 SSRs) in a large number of samples (368 samples) in a short time, confirming that this method has the advantages of higher accuracy regarding genotyping results and shorter time consumption (Table 5). Therefore, the Target SSR-seq method succeeds in providing high-throughput SSR genotyping with high accuracy and efficiency. It not only achieved accurate genotyping of *S. mansoni* populations in the present study, but it also can be widely extended to genetic diversity analyses of other parasites.

**Table 5:** Comparisons of the Target SSR-seq method established in the present study with other existing methods in simple sequence repeat genotyping of parasites

Category	Target SSR-Seq	AmpSeq-SSR^a^	Based on whole genome sequencing^a^	Based on Sanger sequencing	Traditional SSR genotyping techniques		
					Agarose electrophoresis	PAGE electrophoresis	Capillary electrophoresis
Number of PCR cycles	11	16	5–20	40	40	40	40
Slippage possibility^b^	Very low	Low	Low	High	High	High	High
Sequencing depth	> 1000	595	5–10	NA	NA	NA	NA
Experimental procedure	Very simple	Simple	Simple	Simple	Complex	Complex	Simple
Genotyping accuracy	Very high	High	Low	Low	Very low	Low	Low
Time consumption	Short	Short	Short	Very long	Very long	Very long	Very long
Costs	Low	High	Very high	Low	Low	Low	High

*PAGE* Polyacylamide gel electrophoresis

^a^No report of this method being applied to the study of parasites was found, so other fields of study were referred to

^b^The greater number of the PCR cycles, the greater the possibility to incur DNA polymerase slippage

We explored genetic differences among *S. mansoni* isolates from different geographical populations as well as from different host populations. Clustering analysis supported three genetic groups in the 16 geographical populations; one group mainly included populations from central and eastern China, the second group mainly contained populations from southern and southwestern China and the last group only consisted of the FJ population. When considering the detailed genetic diversity of each isolate, although three main clusters were revealed in the phylogenetic tree, no obvious geographical clusters were found, indicating that mixed memberships exist in these geographical populations. In fact, as for most parasitic helminths, the geographical distance is rarely the only determinant of the degree of genetic similarity or difference between populations, and the migration of hosts must also be considered [42]. Both the NJ clustering and PCoA analysis showed that isolates from intermediate hosts (snakes and frogs) tended to cluster together, probably because certain snakes and frogs have similar habitats with overlapping scopes of activities, thus resulting in less geographical isolation and genetic difference of their parasitizing tapeworms [43]. In contrast, parasites isolated from definitive hosts (cats and tigers) have fewer opportunities for gene exchange, thus leading to greater genetic difference. The influence of hosts on parasite genetic polymorphism is likewise limited by other conditions, such as habitat, activity range and whether the migration time of definitive hosts coincides with the period of infection [44]. Therefore, still more numerous factors need to be considered to infer the actual causes of gene exchange and genetic polymorphism of *S. mansoni*.

STRUCTURE analysis divided all *S. mansoni* isolates into two main groups (Pop1 and Pop2), which is consistent with previous studies using multi-gene markers (*cyt*b, *cox*1, *rrn*S, and* 28S* rDNA D1) and traditional SSRs [13, 14]. However, the STRUCTURE analysis did not divide the 16 populations according to their geographical origin; in contrast, each population showed a mixed membership to the inferred clusters. With the exception of isolates from five geographical populations (AH, CQ, HB, JS and SH) that revealed one prominent genotype, the remaining isolates showed two main genotypes. In summary: (i) there are two genotypes in the Chinese *S. mansoni* population, and both genotypes have sympatric distribution in most of geographical populations, with genotype 1 being a prevalent genotype; and (ii) there are two sub-populations: Group 1 and Group 2 are verified, with Group 1 mainly containing isolates from east and central China, and Group 2 containing isolates from south and southwest China.

 The *F*_st_ value was used to measure genetic differentiation among geographical populations [45]. Generally, an *F*_st_ value within the range of 0–0.05 indicates little genetic differentiation; 0.05–0.15, moderate differentiation; 0.15–0.25, large differentiation; and > 0.25, very large genetic differentiation [46]. The pairwise *F*_st_ between Pop1 and Pop2 was estimated at 0.066, indicating moderate genetic differentiation between the two sub-populations. When considering the 16 geographical populations, except for *F*st values between ZJ and AH, ZJ and CQ, ZJ and GZ, SC and GZ, ZJ and HuN, and SC and HuN, respectively, most of the *F*_st_ values were fell within the range of 0.05–0.15, also indicating moderate genetic differentiation. In addition, we observed higher pairwise *F*_st_ values between HN and the other 15 geographical populations, possibly due to the isolation of HN island from the mainland, leading to difficulties in gene exchange. Consistent with the results of estimated *F*_st_, the AMOVA analysis estimated moderate genetic variation of *S. mansoni* in China. Generally, genetic variation among populations is highly affected by genetic drift, gene flow, mutations, selection and long-term evolution [47]. Of the 16 geographical populations identified in this study, five migration events were detected in the TreeMix analysis, namely migration began from GZ to SC, SC to JX, SC to HB, GZ to YN and GZ to JS, indicating that there was some small amount of genetic exchange among the geographical populations. Usually, the populations with frequent gene exchange have a lower degree of genetic differentiation, whereas populations with low frequent gene exchange will exhibit greater genetic differentiation [48]. Gene exchange between isolates of *S. mansoni* is not very frequent, which may also account for the moderate genetic differentiation between populations.

Last but not least, a core set of 23 SSRs were screened further, and a core set of *S. mansoni* samples in each geographical population was successfully generated using the core set of 23 SSRs. Finally, a total of 76 isolates were collected thatcan represent 91.1% genetic diversity of all 362 samples. The establishment of core SSRs and core samples is essential for the optimal management and use of genetic resources of *S. mansoni*, and they provide a strong foundation for establishing core SSRs and core samples for the *Spirometra* species.

## Conclusions

In this study, we successfully established a Target SSR-seq method containing 34 perfect SSR loci for genotyping *S. mansoni* isolates. The Target SSR-seq method provides high-throughput SSR genotyping with high accuracy and efficiency. It enables accurate genotyping of *S. mansoni* populations and can be widely extended to genetic diversity analyses of other parasites. The results of our comprehensive genetic diversity study based on this newly established method suggest that *S. mansoni* samples in China have high genetic diversity and moderate genetic differentiation, and that little genetic exchange exists among geographical populations. In future studies, more populations of *Spirometra* isolates across worldwide geographical ranges are needed to reveal the exact genetic patterns of this medical tapeworm and discover the underlying mechanisms generating such genetic variation patterns.

## Supplementary Information

**Additional file 1: Figure S1. **Estimates of pairwise *F*_st_ between *S. mansoni* populations. **Figure S2. **Estimated genetic structure of *S. mansoni* in China as inferred by the STRUCTURE software on the basis of the data on a core set of 23 SSRs. **Figure S3. **Principal coordinate analysis (PCoA) describing the relationships of 362 *S. mansoni* isolates on the basis of the data on a core set of 23 SSRs. **Table S1. **Information of origin, locality and date of collection for 368 *S. mansoni* isolates. **Table S2. **Primers of cytochrome* c *oxidase subunit 1 (*cox1*) gene. **Table S3. **SSR mining in *S. mansoni*. **Table S4. **Characteristics of a core set of 23 SSR markers. **Table S5.** Analysis of molecular variance (AMOVA) of the populations of *S. mansoni *based on a core set of 23 SSRs. **Table S6. **Core samples of *S. mansoni* identified in each geographical population of China.

## Data Availability

The raw data reported in this study have been deposited in the Genome Sequence Archive1 under accession numbers CRA006630. The data supporting the conclusions of this article are included within the article.

## Abbreviations

AMOVA: Analysis of molecular variance
*cox1*: Cytochrome* c* oxidase subunit 1
NJ: Neighbor-joining
PCoA: Principal coordinate analysis
SSRs: Simple sequence repeats
