# Supplementary material for: Genetic structure of Spirometra mansoni (Cestoda: Diphyllobothriidae) populations in China revealed by a Target SSR-seq method
Source: Parasit Vectors. 2022 Dec 23;15:485. doi: 10.1186/s13071-022-05568-1 (PMC9789593; doi:10.1186/s13071-022-05568-1)
Supplement: Supplementary file 1 — Additional file 1: Figure S1. Estimates of pairwise Fst between S. mansoni populations. Figure S2. Estimated genetic structure of S. mansoni in China as inferred by the STRUCTURE software on the basis of the data on a core set of 23 SSRs. Figure S3. Principal coordinate analysis (PCoA) describing the relationships of 362 S. mansoni isolates on the basis of the data on a core set of 23 SSRs. Table S1. Information of origin, locality and date of collection for 368 S. mansoni isolates. Table S2. Primers of cytochrome c oxidase subunit 1 (cox1) gene. Table S3. SSR mining in S. mansoni. Table S4. Characteristics of a core set of 23 SSR markers. Table S5. Analysis of molecular variance (AMOVA) of the populations of S. mansoni based on a core set of 23 SSRs. Table S6. Core samples of S. mansoni identified in each geographical population of China. [file 13071_2022_5568_MOESM1_ESM.doc]

**SUPPORTING INFORMATION**

**Comprehensive genetic diversity analysis of Spirometra mansoni (Cestoda: Diphyllobothriidae) in China using a Target SSR-seq method**

**Table S1.** Information of origin, locality and date of collection for 368 *S. mansoni* isolates.

**Table S2.** Primers of cytochrome c oxidase subunit 1 (*cox1*) gene.

**Table S3.** SSR mining in *S. mansoni*.

**Table S4.** Characteristics of a core set of 23 SSR markers.

**Table S5.** Analysis of molecular variance (AMOVA) of the populations of *S. mansoni* based on a core set of 23 SSRs.

**Table S6.** Core samples of *S. mansoni* identified in each geographical population of China.

**Figure S1.** Estimates of pairwise *F*_st_ between *S. mansoni* populations.

**Figure S2.** Estimated genetic structure of *S. mansoni* in China as inferred by the STRUCTURE software on the basis of the data on a core set of 23 SSRs.

**Figure S3.** Principal coordinate analysis (PCoA) describing the relationships of 362 *S. mansoni* isolates on the basis of the data on a core set of 23 SSRs.

**Table S1.** Information of origin, locality and date of collection for 368 *S. mansoni* isolates.

| **Sample code** | **Host origin** | **Geographical origin** | **Longitude** | **Latitude** | **Collection date** |
| --- | --- | --- | --- | --- | --- |
| AH-HF-1 | *P. nigromaculatus* | Hefei City, Anhui | 117.25 E | 31.88 N | 11 Jul, 2014 |
| AH-HF-2 | *P. nigromaculatus* | Hefei City, Anhui | 117.25 E | 31.88 N | 11 Jul, 2014 |
| AH-HF-3 | *P. nigromaculatus* | Hefei City, Anhui | 117.25 E | 31.88 N | 11 Jul, 2014 |
| AH-HF-4 | *P. nigromaculatus* | Hefei City, Anhui | 117.25 E | 31.88 N | 11 Jul, 2014 |
| AH-HF-5 | *S. latouchii* | Hefei City, Anhui | 117.25 E | 31.88 N | 11 Jul, 2014 |
| AH-HF-6 | *F. limnocharis* | Hefei City, Anhui | 117.25 E | 31.88 N | 11 Jul, 2014 |
| AH-LA-1 | *P. nigromaculatus* | Luan City, Anhui | 116.28 E | 32.35 N | 17 Jul, 2016 |
| AH-LA-2 | *P. nigromaculatus* | Luan City, Anhui | 116.28 E | 32.35 N | 17 Jul, 2016 |
| AH-LA-3 | *P. nigromaculatus* | Luan City, Anhui | 116.28 E | 32.35 N | 17 Jul, 2016 |
| AH-LA-4 | *P. nigromaculatus* | Luan City, Anhui | 116.28 E | 32.35 N | 17 Jul, 2016 |
| AH-LA-5 | *S. latouchii* | Luan City, Anhui | 116.28 E | 32.35 N | 17 Jul, 2016 |
| AH-LA-6 | *S. latouchii* | Luan City, Anhui | 116.28 E | 32.35 N | 17 Jul, 2016 |
| AH-LA-7 | *F. limnocharis* | Luan City, Anhui | 116.28 E | 32.35 N | 17 Jul, 2016 |
| AH-LA-8 | *F. limnocharis* | Luan City, Anhui | 116.28 E | 32.35 N | 17 Jul, 2016 |
| AH-WH-2 | *P. nigromaculatus* | Wuhu City, Anhui | 118.57 E | 31.15 N | 16 Jul, 2014 |
| AH-WH-3 | *F. limnocharis* | Wuhu City, Anhui | 118.57 E | 31.15 N | 16 Jul, 2014 |
| AH-YC-1 | *P. nigromaculatus* | Yicheng City, Anhui | 118.75 E | 30.95 N | 14 Jul, 2014 |
| AH-YC-2 | *S. latouchii* | Yicheng City, Anhui | 118.75 E | 30.95 N | 14 Jul, 2014 |
| CQ-FL-1 | *P. nigromaculatus* | Fuling City, Chongqing | 107.15 E | 29.71 N | 17 Aug, 2018 |
| CQ-FL-2 | *P. nigromaculatus* | Fuling City, Chongqing | 107.15 E | 29.71 N | 17 Aug, 2018 |
| CQ-FL-3 | *O. margaretae* | Fuling City, Chongqing | 107.15 E | 29.71 N | 17 Aug, 2018 |
| CQ-LP-1 | *P. nigromaculatus* | Liangping City, Chongqing | 107.80 E | 30.68 N | 16 Jul, 2014 |
| CQ-LP-2 | *P. nigromaculatus* | Liangping City, Chongqing | 107.80 E | 30.68 N | 16 Jul, 2014 |
| CQ-LP-3 | *P. nigromaculatus* | Liangping City, Chongqing | 107.80 E | 30.68 N | 16 Jul, 2014 |
| CQ-LP-4 | *F. limnocharis* | Liangping City, Chongqing | 107.80 E | 30.68 N | 16 Jul, 2014 |
| CQ-LP-5 | *O. margaretae* | Liangping City, Chongqing | 107.80 E | 30.68 N | 16 Jul, 2014 |
| CQ-YoY-1 | *P. nigromaculatus* | Youyang City, Chongqing | 108.96 E | 28.90 N | 21 Aug, 2018 |
| CQ-YoY-2 | *P. nigromaculatus* | Youyang City, Chongqing | 108.96 E | 28.90 N | 21 Aug, 2018 |
| CQ-YY-1 | *P. nigromaculatus* | Yueyang City, Chongqing | 108.70 E | 30.93 N | 2 Aug, 2018 |
| CQ-YY-2 | *P. nigromaculatus* | Yueyang City, Chongqing | 108.70 E | 30.93 N | 2 Aug, 2018 |
| CQ-YY-3 | *P. nigromaculatus* | Yueyang City, Chongqing | 108.70 E | 30.93 N | 2 Aug, 2018 |
| CQ-YY-4 | *F. limnocharis* | Yueyang City, Chongqing | 108.70 E | 30.93 N | 2 Aug, 2018 |
| CQ-YY-5 | *F. limnocharis* | Yueyang City, Chongqing | 108.70 E | 30.93 N | 2 Aug, 2018 |
| CQ-YY-6 | *F. limnocharis* | Yueyang City, Chongqing | 108.70 E | 30.93 N | 2 Aug, 2018 |
| CQ-YY-7 | *O. margaretae* | Yueyang City, Chongqing | 108.70 E | 30.93 N | 2 Aug, 2018 |
| CQ-YY-8 | *O. margaretae* | Yueyang City, Chongqing | 108.70 E | 30.93 N | 2 Aug, 2018 |
| FJ-ND-1 | *P. nigromaculatus* | Ningde City, Fujian | 119.51 E | 27.45 N | 21 Jul, 2016 |
| FJ-ND-2 | *P. nigromaculatus* | Ningde City, Fujian | 119.51 E | 27.45 N | 21 Jul, 2016 |
| FJ-ND-3 | *S. latouchii* | Ningde City, Fujian | 119.51 E | 27.45 N | 21 Jul, 2016 |
| FJ-NP-1 | *P. nigromaculatus* | Nanping City, Fujian | 118.52 E | 26.63 N | 24 Jul, 2016 |
| FJ-NP-2 | *P. nigromaculatus* | Nanping City, Fujian | 118.52 E | 26.63 N | 24 Jul, 2016 |
| FJ-NP-3 | *S. latouchii* | Nanping City, Fujian | 118.52 E | 26.63 N | 24 Jul, 2016 |
| FJ-QZ-1 | *P. nigromaculatus* | Quanzhou City, Fujian | 118.68 E | 24.87 N | 10 Jul, 2017 |
| FJ-QZ-2 | *P. nigromaculatus* | Quanzhou City, Fujian | 118.68 E | 24.87 N | 10 Jul, 2017 |
| FJ-QZ-3 | *S. latouchii* | Quanzhou City, Fujian | 118.68 E | 24.87 N | 10 Jul, 2017 |
| FJ-QZ-4 | *S. latouchii* | Quanzhou City, Fujian | 118.68 E | 24.87 N | 10 Jul, 2017 |
| GD-DG-1 | *P. nigromaculatus* | Dongguan City, Guangdong | 113.87 E | 23.08 N | 27 Jul, 2014 |
| GD-DG-2 | *P. nigromaculatus* | Dongguan City, Guangdong | 113.87 E | 23.08 N | 27 Jul, 2014 |
| GD-DG-3 | *S. latouchii* | Dongguan City, Guangdong | 113.87 E | 23.08 N | 27 Jul, 2014 |
| GD-FS-1 | *P. nigromaculatus* | Fushan City, Guangdong | 113.29 E | 22.81 N | 17 Aug, 2014 |
| GD-FS-2 | *P. nigromaculatus* | Fushan City, Guangdong | 113.29 E | 22.81 N | 17 Aug, 2014 |
| GD-FS-10 | *S. latouchii* | Fushan City, Guangdong | 113.29 E | 22.81 N | 17 Aug, 2014 |
| GD-FS-3 | *P. nigromaculatus* | Fushan City, Guangdong | 113.29 E | 22.81 N | 17 Aug, 2014 |
| GD-FS-4 | *P. nigromaculatus* | Fushan City, Guangdong | 113.29 E | 22.81 N | 17 Aug, 2014 |
| GD-FS-5 | *P. nigromaculatus* | Fushan City, Guangdong | 113.29 E | 22.81 N | 17 Aug, 2014 |
| GD-FS-6 | *P. nigromaculatus* | Fushan City, Guangdong | 113.29 E | 22.81 N | 17 Aug, 2014 |
| GD-FS-7 | *P. nigromaculatus* | Fushan City, Guangdong | 113.29 E | 22.81 N | 17 Aug, 2014 |
| GD-FS-8 | *S. latouchii* | Fushan City, Guangdong | 113.29 E | 22.81 N | 17 Aug, 2014 |
| GD-FS-9 | *S. latouchii* | Fushan City, Guangdong | 113.29 E | 22.81 N | 17 Aug, 2014 |
| GD-GZ-1 | *P. nigromaculatus* | Guangzhou City, Guangdong | 113.26 E | 23.13 N | 18 Jul, 2015 |
| GD-GZ-2 | *P. nigromaculatus* | Guangzhou City, Guangdong | 113.26 E | 23.13 N | 18 Jul, 2015 |
| GD-JM-2 | *P. nigromaculatus* | Jiangmen City, Guangdong | 113.09 E | 22.59 N | 22 Jul, 2017 |
| GD-JM-3 | *P. nigromaculatus* | Jiangmen City, Guangdong | 113.09 E | 22.59 N | 22 Jul, 2017 |
| GD-JM-5 | *S. latouchii* | Jiangmen City, Guangdong | 113.09 E | 22.59 N | 22 Jul, 2017 |
| GX-GL-1 | *P. nigromaculatus* | Guilin City, Guangxi | 110.28 E | 25.29 N | 30 Aug, 2013 |
| GX-GL-2 | *P. nigromaculatus* | Guilin City, Guangxi | 110.28 E | 25.29 N | 30 Aug, 2013 |
| GX-GL-3 | *O. margaretae* | Guilin City, Guangxi | 110.28 E | 25.29 N | 30 Aug, 2013 |
| GX-GL-4 | *S. latouchii* | Guilin City, Guangxi | 110.28 E | 25.29 N | 30 Aug, 2013 |
| GX-NN-1 | *P. nigromaculatus* | Nanning City, Guangxi | 108.21 E | 22.51 N | 9 Aug, 2013 |
| GX-NN-2 | *P. nigromaculatus* | Nanning City, Guangxi | 108.21 E | 22.51 N | 9 Aug, 2013 |
| GX-NN-3 | *P. nigromaculatus* | Nanning City, Guangxi | 108.21 E | 22.51 N | 9 Aug, 2013 |
| GX-NN-4 | *B. guentheri* | Nanning City, Guangxi | 108.21 E | 22.51 N | 9 Aug, 2013 |
| GX-NN-5 | *S. latouchii* | Nanning City, Guangxi | 108.21 E | 22.51 N | 9 Aug, 2013 |
| GX-NN-6 | *S. latouchii* | Nanning City, Guangxi | 108.21 E | 22.51 N | 9 Aug, 2013 |
| GX-WZ-1 | *P. nigromaculatus* | Wuzhou City, Guangxi | 111.54 E | 23.85 N | 18 Jul, 2016 |
| GX-WZ-10 | *B. guentheri* | Wuzhou City, Guangxi | 111.54 E | 23.85 N | 18 Jul, 2016 |
| GX-WZ-11 | *P. nigromaculatus* | Wuzhou City, Guangxi | 111.54 E | 23.85 N | 18 Jul, 2016 |
| GX-WZ-2 | *P. nigromaculatus* | Wuzhou City, Guangxi | 111.54 E | 23.85 N | 18 Jul, 2016 |
| GX-WZ-3 | *P. nigromaculatus* | Wuzhou City, Guangxi | 111.54 E | 23.85 N | 18 Jul, 2016 |
| GX-WZ-4 | *P. nigromaculatus* | Wuzhou City, Guangxi | 111.54 E | 23.85 N | 18 Jul, 2016 |
| GX-WZ-5 | *S. latouchii* | Wuzhou City, Guangxi | 111.54 E | 23.85 N | 18 Jul, 2016 |
| GX-WZ-6 | *O. margaretae* | Wuzhou City, Guangxi | 111.54 E | 23.85 N | 18 Jul, 2016 |
| GX-WZ-7 | *B. guentheri* | Wuzhou City, Guangxi | 111.54 E | 23.85 N | 18 Jul, 2016 |
| GX-WZ-8 | *S. latouchii* | Wuzhou City, Guangxi | 111.54 E | 23.85 N | 18 Jul, 2016 |
| GX-WZ-9 | *S. latouchii* | Wuzhou City, Guangxi | 111.54 E | 23.85 N | 18 Jul, 2016 |
| GX-YL-1 | *P. nigromaculatus* | Yulin City, Guangxi | 110.16 E | 22.19 N | 16 Jul, 2013 |
| GX-YL-2 | *P. nigromaculatus* | Yulin City, Guangxi | 110.16 E | 22.19 N | 16 Jul, 2013 |
| GX-YL-3 | *S. latouchii* | Yulin City, Guangxi | 110.16 E | 22.19 N | 16 Jul, 2013 |
| GZ-AS-1 | *P. nigromaculatus* | Anshun City, Guizhou | 105.95 E | 26.25 N | 7 Aug, 2013 |
| GZ-AS-2 | *P. nigromaculatus* | Anshun City, Guizhou | 105.95 E | 26.25 N | 7 Aug, 2013 |
| GZ-GY-1 | *P. nigromaculatus* | Guiyang City, Guizhou | 106.63 E | 26.65 N | 2 Aug, 2013 |
| GZ-GY-2 | *P. nigromaculatus* | Guiyang City, Guizhou | 106.63 E | 26.65 N | 2 Aug, 2013 |
| GZ-GY-3 | *P. nigromaculatus* | Guiyang City, Guizhou | 106.63 E | 26.65 N | 2 Aug, 2013 |
| GZ-GY-4 | *S. latouchii* | Guiyang City, Guizhou | 106.63 E | 26.65 N | 2 Aug, 2013 |
| GZ-GY-5 | *O. margaretae* | Guiyang City, Guizhou | 106.63 E | 26.65 N | 2 Aug, 2013 |
| GZ-GY-6 | *O. margaretae* | Guiyang City, Guizhou | 106.63 E | 26.65 N | 2 Aug, 2013 |
| GZ-KL-1 | *P. nigromaculatus* | Kaili City, Guizhou | 107.63 E | 26.53 N | 11 Jul, 2014 |
| GZ-KL-2 | *P. nigromaculatus* | Kaili City, Guizhou | 107.63 E | 26.53 N | 11 Jul, 2014 |
| GZ-KL-3 | *P. nigromaculatus* | Kaili City, Guizhou | 107.63 E | 26.53 N | 11 Jul, 2014 |
| GZ-KL-4 | *P. nigromaculatus* | Kaili City, Guizhou | 107.63 E | 26.53 N | 11 Jul, 2014 |
| GZ-KL-5 | *P. nigromaculatus* | Kaili City, Guizhou | 107.63 E | 26.53 N | 11 Jul, 2014 |
| GZ-KL-6 | *O. margaretae* | Kaili City, Guizhou | 107.63 E | 26.53 N | 11 Jul, 2014 |
| GZ-KL-7 | *B. guentheri* | Kaili City, Guizhou | 107.63 E | 26.53 N | 11 Jul, 2014 |
| GZ-KL-8 | *S. latouchii* | Kaili City, Guizhou | 107.63 E | 26.53 N | 11 Jul, 2014 |
| GZ-KL-9 | *S. latouchii* | Kaili City, Guizhou | 107.63 E | 26.53 N | 11 Jul, 2014 |
| GZ-ZY-1 | *P. nigromaculatus* | Zunyi City, Guizhou | 107.45 E | 28.55 N | 30 Jul, 2014 |
| GZ-ZY-2 | *P. nigromaculatus* | Zunyi City, Guizhou | 107.45 E | 28.55 N | 30 Jul, 2014 |
| GZ-ZY-3 | *P. nigromaculatus* | Zunyi City, Guizhou | 107.45 E | 28.55 N | 30 Jul, 2014 |
| GZ-ZY-4 | *S. latouchii* | Zunyi City, Guizhou | 107.45 E | 28.55 N | 30 Jul, 2014 |
| HB-HG-1 | *P. nigromaculatus* | Huanggang City, Hubei | 114.88 E | 30.45 N | 28 Jul, 2016 |
| HB-HG-2 | *P. nigromaculatus* | Huanggang City, Hubei | 114.88 E | 30.45 N | 28 Jul, 2016 |
| HB-HG-3 | *P. nigromaculatus* | Huanggang City, Hubei | 114.88 E | 30.45 N | 28 Jul, 2016 |
| HB-HG-4 | *S. latouchii* | Huanggang City, Hubei | 114.88 E | 30.45 N | 28 Jul, 2016 |
| HB-HG-5 | *B. guentheri* | Huanggang City, Hubei | 114.88 E | 30.45 N | 28 Jul, 2016 |
| HB-HG-6 | *F. limnocharis* | Huanggang City, Hubei | 114.88 E | 30.45 N | 28 Jul, 2016 |
| HB-HG-7 | *F. limnocharis* | Huanggang City, Hubei | 114.88 E | 30.45 N | 28 Jul, 2016 |
| HB-HG-8 | *F. limnocharis* | Huanggang City, Hubei | 114.88 E | 30.45 N | 28 Jul, 2016 |
| HB-XG-1 | *P. nigromaculatus* | Xiaogan City, Hubei | 113.75 E | 31.02 N | 16 Jul, 2015 |
| HB-XG-2 | *P. nigromaculatus* | Xiaogan City, Hubei | 113.75 E | 31.02 N | 16 Jul, 2015 |
| HB-XG-4 | *P. nigromaculatus* | Xiaogan City, Hubei | 113.75 E | 31.02 N | 16 Jul, 2015 |
| HB-XG-5 | *S. latouchii* | Xiaogan City, Hubei | 113.75 E | 31.02 N | 16 Jul, 2015 |
| HB-XG-6 | *F. limnocharis* | Xiaogan City, Hubei | 113.75 E | 31.02 N | 16 Jul, 2015 |
| HB-XG-7 | *B. guentheri* | Xiaogan City, Hubei | 113.75 E | 31.02 N | 16 Jul, 2015 |
| HB-XG-8 | *B. guentheri* | Xiaogan City, Hubei | 113.75 E | 31.02 N | 16 Jul, 2015 |
| HB-XN-1 | *P. nigromaculatus* | Xianning City, Hubei | 114.04 E | 29.56 N | 15 Jul, 2015 |
| HB-XN-2 | *P. nigromaculatus* | Xianning City, Hubei | 114.04 E | 29.56 N | 15 Jul, 2015 |
| HeN-LH-1 | *P. nigromaculatus* | Luohe City, Henan | 114.02 E | 33.58 N | 24 Jul, 2013 |
| HeN-LH-2 | *P. nigromaculatus* | Luohe City, Henan | 114.02 E | 33.58 N | 24 Jul, 2013 |
| HeN-LH-3 | *B. guentheri* | Luohe City, Henan | 114.02 E | 33.58 N | 24 Jul, 2013 |
| HeN-LH-4 | *F. limnocharis* | Luohe City, Henan | 114.02 E | 33.58 N | 24 Jul, 2013 |
| HeN-LH-5 | *F. limnocharis* | Luohe City, Henan | 114.02 E | 33.58 N | 24 Jul, 2013 |
| HeN-LH-6 | *F. limnocharis* | Luohe City, Henan | 114.02 E | 33.58 N | 24 Jul, 2013 |
| HeN-NY-1 | *P. nigromaculatus* | Nanyang City, Henan | 112.43 E | 33.49 N | 7 Aug, 2018 |
| HeN-NY-2 | *B. guentheri* | Nanyang City, Henan | 112.43 E | 33.49 N | 7 Aug, 2018 |
| HeN-NY-3 | *F. limnocharis* | Nanyang City, Henan | 112.43 E | 33.49 N | 7 Aug, 2018 |
| HeN-NY-4 | *F. limnocharis* | Nanyang City, Henan | 112.43 E | 33.49 N | 7 Aug, 2018 |
| HeN-XX-1 | *P. nigromaculatus* | Xinxiang City, Henan | 113.87 E | 35.30 N | 7 Aug, 2013 |
| HeN-XX-2 | *P. nigromaculatus* | Xinxiang City, Henan | 113.87 E | 35.30 N | 7 Aug, 2013 |
| HeN-XY-1 | *F. limnocharis* | Xinyang City, Henan | 114.06 E | 32.10 N | 12 Aug, 2018 |
| HeN-XY-2 | *F. limnocharis* | Xinyang City, Henan | 114.06 E | 32.10 N | 12 Aug, 2018 |
| HeN-ZK-1 | *P. nigromaculatus* | Zhoukou City, Henan | 114.38 E | 34.07 N | 17 Aug, 2013 |
| HeN-ZK-2 | *F. limnocharis* | Zhoukou City, Henan | 114.38 E | 34.07 N | 17 Aug, 2013 |
| HeN-ZZ-1 | *P. nigromaculatus* | Zhengzhou City, Henan | 113.65 E | 34.73 N | 30 Aug, 2013 |
| HeN-ZZ-2 | *P. nigromaculatus* | Zhengzhou City, Henan | 113.65 E | 34.73 N | 30 Aug, 2013 |
| HeN-ZZ-3 | *P. nigromaculatus* | Zhengzhou City, Henan | 113.65 E | 34.73 N | 30 Aug, 2013 |
| HeN-ZZ-4 | *P. nigromaculatus* | Zhengzhou City, Henan | 113.65 E | 34.73 N | 30 Aug, 2013 |
| HeN-ZZ-5 | *F. limnocharis* | Zhengzhou City, Henan | 113.65 E | 34.73 N | 30 Aug, 2013 |
| HN-HK-1 | *P. nigromaculatus* | Haikou City, Hainan | 110.37 E | 20.03 N | 30 Jul, 2013 |
| HN-HK-2 | *P. nigromaculatus* | Haikou City, Hainan | 110.37 E | 20.03 N | 30 Jul, 2013 |
| HN-WZS-1 | *P. nigromaculatus* | Wuzhishan City, Hainan | 110.40 E | 18.80 N | 17 Jul, 2013 |
| HN-WZS-4 | *P. nigromaculatus* | Wuzhishan City, Hainan | 110.40 E | 18.80 N | 17 Jul, 2013 |
| HN-WZS-5 | *P. nigromaculatus* | Wuzhishan City, Hainan | 110.40 E | 18.80 N | 17 Jul, 2013 |
| HN-WZS-6 | *P. nigromaculatus* | Wuzhishan City, Hainan | 110.40 E | 18.80 N | 17 Jul, 2013 |
| HN-WZS-7 | *B. guentheri* | Wuzhishan City, Hainan | 110.40 E | 18.80 N | 17 Jul, 2013 |
| HN-WZS-8 | *B. guentheri* | Wuzhishan City, Hainan | 110.40 E | 18.80 N | 17 Jul, 2013 |
| HN-WZS-9 | *B. guentheri* | Wuzhishan City, Hainan | 110.40 E | 18.80 N | 17 Jul, 2013 |
| HuN-CS-1 | *P. nigromaculatus* | Changsha City, Hunan | 113.04 E | 28.14 N | 26 Jun, 2013 |
| HuN-CS-2 | *P. nigromaculatus* | Changsha City, Hunan | 113.04 E | 28.14 N | 26 Jun, 2013 |
| HuN-CS-3 | *Z. dhumnades* | Changsha City, Hunan | 112.57 E | 28.27 N | 2019 |
| HuN-CS-4 | *Z. dhumnades* | Changsha City, Hunan | 112.57 E | 28.27 N | 2019 |
| HuN-CS-5 | *P. tigris tigris* | Changsha City, Hunan | 112.57 E | 28.27 N | 2019 |
| HuN-CS-6 | *P. tigris tigris* | Changsha City, Hunan | 112.57 E | 28.27 N | 2019 |
| HuN-CS-7 | *P. tigris altaica* | Changsha City, Hunan | 112.57 E | 28.27 N | 2019 |
| HuN-CS-8 | *P. tigris altaica* | Changsha City, Hunan | 112.57 E | 28.27 N | 2019 |
| HuN-CS-9 | *P. bengalensis* | Changsha City, Hunan | 112.57 E | 28.27 N | 2019 |
| HuN-CS-10 | *P. bengalensis* | Changsha City, Hunan | 112.57 E | 28.27 N | 2019 |
| HuN-CS-11 | *F. catus* | Changsha City, Hunan | 112.57 E | 28.27 N | 2019 |
| HuN-CS-12 | *F. catus* | Changsha City, Hunan | 112.57 E | 28.27 N | 2019 |
| HuN-HH-1 | *P. nigromaculatus* | Huaihua City, Hunan | 110.59 E | 27.91 N | 30 Jul, 2015 |
| HuN-HH-2 | *P. nigromaculatus* | Huaihua City, Hunan | 110.59 E | 27.91 N | 30 Jul, 2015 |
| HuN-HH-3 | *S. latouchii* | Huaihua City, Hunan | 110.59 E | 27.91 N | 30 Jul, 2015 |
| HuN-HH-4 | *Z. dhumnades* | Huaihua City, Hunan | 110.59 E | 27.91 N | 2019 |
| HuN-HH-5 | *Z. dhumnades* | Huaihua City, Hunan | 110.59 E | 27.91 N | 2019 |
| HuN-HY-1 | *P. nigromaculatus* | Hengyang City, Hunan | 112.83 E | 26.31 N | 3 Aug, 2016 |
| HuN-HY-2 | *P. nigromaculatus* | Hengyang City, Hunan | 112.83 E | 26.31 N | 3 Aug, 2016 |
| HuN-HY-3 | *P. nigromaculatus* | Hengyang City, Hunan | 112.83 E | 26.31 N | 3 Aug, 2016 |
| HuN-HY-4 | *P. nigromaculatus* | Hengyang City, Hunan | 112.83 E | 26.31 N | 3 Aug, 2016 |
| HuN-JS-1 | *P. nigromaculatus* | Jishou City, Hunan | 109.73 E | 28.32 N | 2019 |
| HuN-JS-2 | *P. nigromaculatus* | Jishou City, Hunan | 109.73 E | 28.32 N | 2019 |
| HuN-JS-3 | *B. guentheri* | Jishou City, Hunan | 109.73 E | 28.32 N | 2019 |
| HuN-SY-1 | *P. nigromaculatus* | Shaoyang City, Hunan | 111.74 E | 27.26 N | 9 Aug, 2015 |
| HuN-SY-2 | *P. nigromaculatus* | Shaoyang City, Hunan | 111.74 E | 27.26 N | 9 Aug, 2015 |
| HuN-SY-3 | *Z. dhumnades* | Shaoyang City, Hunan | 116.39 E | 39.92 N | 2019 |
| HuN-SY-4 | *Z. dhumnades* | Shaoyang City, Hunan | 116.39 E | 39.92 N | 2019 |
| HuN-SY-5 | *E. carinata* | Shaoyang City, Hunan | 116.39 E | 39.92 N | 2019 |
| HuN-SY-6 | *E. carinata* | Shaoyang City, Hunan | 116.39 E | 39.92 N | 2019 |
| HuN-XXfh-1 | *P. nigromaculatus* | Xiangxi City, Hunan | 109.58 E | 27.96 N | 8 Aug, 2016 |
| HuN-XX-2 | *P. nigromaculatus* | Xiangxi City, Hunan | 109.58 E | 27.96 N | 8 Aug, 2016 |
| HuN-XXfh-4 | *S. latouchii* | Xiangxi City, Hunan | 109.58 E | 27.96 N | 8 Aug, 2016 |
| HuN-XXfh-5 | *B. guentheri* | Xiangxi City, Hunan | 109.58 E | 27.96 N | 8 Aug, 2016 |
| HuN-XXfh-6 | *B. guentheri* | Xiangxi City, Hunan | 109.58 E | 27.96 N | 8 Aug, 2016 |
| HuN-XiX-1 | *Z. dhumnades* | Xiangxi City, Hunan | 109.74 E | 28.31 N | 2019 |
| HuN-XiX-2 | *Z. dhumnades* | Xiangxi City, Hunan | 109.74 E | 28.31 N | 2019 |
| HuN-YY-1 | *P. nigromaculatus* | Yueyang City, Hunan | 112.54 E | 29.53 N | 28 Jul, 2015 |
| HuN-YY-2 | *P. nigromaculatus* | Yueyang City, Hunan | 112.54 E | 29.53 N | 28 Jul, 2015 |
| HuN-YY-3 | *P. nigromaculatus* | Yueyang City, Hunan | 112.54 E | 29.53 N | 28 Jul, 2015 |
| HuN-YY-4 | *B. guentheri* | Yueyang City, Hunan | 112.54 E | 29.53 N | 28 Jul, 2015 |
| HuN-YY-5 | *Z. dhumnades* | Yueyang City, Hunan | 113.00 E | 29.46 N | 2019 |
| HuN-YY-6 | *Z. dhumnades* | Yueyang City, Hunan | 113.00 E | 29.46 N | 2019 |
| HuN-YY-7 | *E. carinata* | Yueyang City, Hunan | 113.00 E | 29.46 N | 2019 |
| HuN-YY-8 | *E. carinata* | Yueyang City, Hunan | 113.00 E | 29.46 N | 2019 |
| HuN-ZJJ-1 | *P. nigromaculatus* | Zhangjiajie City, Hunan | 110.20 E | 29.41 N | 19 Jul, 2015 |
| HuN-ZJJ-2 | *P. nigromaculatus* | Zhangjiajie City, Hunan | 110.20 E | 29.41 N | 19 Jul, 2015 |
| HuN-ZJJ-3 | *S. latouchii* | Zhangjiajie City, Hunan | 110.20 E | 29.41 N | 19 Jul, 2015 |
| HuN-ZJJ-4 | *B. guentheri* | Zhangjiajie City, Hunan | 110.20 E | 29.41 N | 19 Jul, 2015 |
| HuN-ZJJ-5 | *Z. dhumnades* | Zhangjiajie City, Hunan | 110.55 E | 29.35 N | 2019 |
| HuN-ZJJ-6 | *Z. dhumnades* | Zhangjiajie City, Hunan | 110.55 E | 29.35 N | 2019 |
| HuN-YiY-1 | *Z. dhumnades* | Yiyang City, Hunan | 112.37 E | 28.57 N | 2019 |
| HuN-YiY-2 | *Z. dhumnades* | Yiyang City, Hunan | 112.37 E | 28.57 N | 2019 |
| HuN-YiY-3 | *E. carinata* | Yiyang City, Hunan | 112.37 E | 28.57 N | 2019 |
| HuN-YiY-4 | *E. carinata* | Yiyang City, Hunan | 112.37 E | 28.57 N | 2019 |
| HuN-CD-1 | *Z. dhumnades* | Changde City, Hunan | 111.68 E | 29.02 N | 2019 |
| HuN-CD-2 | *Z. dhumnades* | Changde City, Hunan | 111.68 E | 29.02 N | 2019 |
| HuN-CD-3 | *E. carinata* | Changde City, Hunan | 111.68 E | 29.02 N | 2019 |
| HuN-CD-4 | *E. carinata* | Changde City, Hunan | 111.68 E | 29.02 N | 2019 |
| HuN-YZ-1 | *Z. dhumnades* | Yongzhou City, Hunan | 111.59 E | 26.46 N | 2019 |
| HuN-YZ-2 | *Z. dhumnades* | Yongzhou City, Hunan | 111.59 E | 26.46 N | 2019 |
| HuN-HY-5 | *Z. dhumnades* | Hengyang City, Hunan | 112.85 E | 26.42 N | 2019 |
| HuN-HY-6 | *Z. dhumnades* | Hengyang City, Hunan | 112.85 E | 26.42 N | 2019 |
| HuN-HY-7 | *E. taeniura* | Hengyang City, Hunan | 112.85 E | 26.42 N | 2019 |
| HuN-HY-8 | *E. taeniura* | Hengyang City, Hunan | 112.85 E | 26.42 N | 2019 |
| HuN-XT-1 | *Z. dhumnades* | Xiangtan City, Hunan | 112.90 E | 27.85 N | 2019 |
| HuN-XT-2 | *Z. dhumnades* | Xiangtan City, Hunan | 112.90 E | 27.85 N | 2019 |
| HuN-XT-3 | *E. carinata* | Xiangtan City, Hunan | 112.90 E | 27.85 N | 2019 |
| HuN-XT-4 | *E. carinata* | Xiangtan City, Hunan | 112.90 E | 27.85 N | 2019 |
| HuN-ZZ-1 | *Z. dhumnades* | Zhuzhou City, Hunan | 112.53 E | 27.73 N | 2019 |
| HuN-ZZ-2 | *Z. dhumnades* | Zhuzhou City, Hunan | 112.53 E | 27.73 N | 2019 |
| HuN-ZZ-3 | *E. taeniura* | Zhuzhou City, Hunan | 112.53 E | 27.73 N | 2019 |
| HuN-ZZ-4 | *E. taeniura* | Zhuzhou City, Hunan | 112.53 E | 27.73 N | 2019 |
| HuN-LD-1 | *E. carinata* | Loudi City, Hunan | 112.00 E | 27.72 N | 2019 |
| HuN-LD-2 | *E. carinata* | Loudi City, Hunan | 112.00 E | 27.72 N | 2019 |
| HuN-CZ-1 | *Z. dhumnades* | Chenzhou City, Hunan | 113.01 E | 25.78 N | 2019 |
| HuN-CZ-2 | *Z. dhumnades* | Chenzhou City, Hunan | 113.01 E | 25.78 N | 2019 |
| JS-SZ-1 | *P. nigromaculatus* | Suzhou City, Jiangsu | 120.98 E | 31.38 N | 8 Jul, 2014 |
| JS-SZ-2 | *P. nigromaculatus* | Suzhou City, Jiangsu | 120.98 E | 31.38 N | 8 Jul, 2014 |
| JS-SZ-4 | *P. nigromaculatus* | Suzhou City, Jiangsu | 120.98 E | 31.38 N | 8 Jul, 2014 |
| JS-SZ-5 | *S. latouchii* | Suzhou City, Jiangsu | 120.98 E | 31.38 N | 8 Jul, 2014 |
| JS-SZ-6 | *F. limnocharis* | Suzhou City, Jiangsu | 120.98 E | 31.38 N | 8 Jul, 2014 |
| JS-SZ-7 | *F. limnocharis* | Suzhou City, Jiangsu | 120.98 E | 31.38 N | 8 Jul, 2014 |
| JS-YC-1 | *P. nigromaculatus* | Yancheng City, Jiangsu | 119.80 E | 33.78 N | 14 Jul, 2014 |
| JS-YC-2 | *S. latouchii* | Yancheng City, Jiangsu | 119.80 E | 33.78 N | 14 Jul, 2014 |
| JS-ZJ-1 | *P. nigromaculatus* | Zhenjiang City, Jiangsu | 119.41 E | 32.20 N | 7 Jul, 2014 |
| JS-ZJ-2 | *P. nigromaculatus* | Zhenjiang City, Jiangsu | 119.41 E | 32.20 N | 7 Jul, 2014 |
| JS-ZJ-3 | *P. nigromaculatus* | Zhenjiang City, Jiangsu | 119.41 E | 32.20 N | 7 Jul, 2014 |
| JS-ZJ-4 | *P. nigromaculatus* | Zhenjiang City, Jiangsu | 119.41 E | 32.20 N | 7 Jul, 2014 |
| JS-ZJ-5 | *P. nigromaculatus* | Zhenjiang City, Jiangsu | 119.41 E | 32.20 N | 7 Jul, 2014 |
| JS-ZJ-6 | *S. latouchii* | Zhenjiang City, Jiangsu | 119.41 E | 32.20 N | 7 Jul, 2014 |
| JS-ZJ-7 | *S. latouchii* | Zhenjiang City, Jiangsu | 119.41 E | 32.20 N | 7 Jul, 2014 |
| JS-ZJ-8 | *F. limnocharis* | Zhenjiang City, Jiangsu | 119.41 E | 32.20 N | 7 Jul, 2014 |
| JS-ZJ-9 | *F. limnocharis* | Zhenjiang City, Jiangsu | 119.41 E | 32.20 N | 7 Jul, 2014 |
| JX-FZcg-2 | *P. nigromaculatus* | Fuzhou City, Jiangsu | 116.38 E | 27.9 N | 15 Jul, 2014 |
| JX-FZcg-3 | *P. nigromaculatus* | Fuzhou City, Jiangsu | 116.38 E | 27.9 N | 15 Jul, 2014 |
| JX-FZcg-4 | *P. nigromaculatus* | Fuzhou City, Jiangsu | 116.38 E | 27.9 N | 15 Jul, 2014 |
| JX-FZcg-5 | *P. nigromaculatus* | Fuzhou City, Jiangsu | 116.38 E | 27.90 N | 15 Jul, 2014 |
| JX-FZcg-6 | *F. limnocharis* | Fuzhou City, Jiangsu | 116.38 E | 27.90 N | 15 Jul, 2014 |
| JX-FZcr-1 | *P. nigromaculatus* | Fuzhou City, Jiangsu | 116.06 E | 27.77 N | 15 Aug, 2014 |
| JX-FZcr-2 | *P. nigromaculatus* | Fuzhou City, Jiangsu | 116.06 E | 27.77 N | 15 Aug, 2014 |
| JX-FZlc-1 | *P. nigromaculatus* | Fuzhou City, Jiangsu | 116.31 E | 27.93 N | 8 Jul, 2014 |
| JX-FZlc-2 | *P. nigromaculatus* | Fuzhou City, Jiangsu | 116.31 E | 27.93 N | 8 Jul, 2014 |
| JX-FZlc-3 | *P. nigromaculatus* | Fuzhou City, Jiangsu | 116.31 E | 27.93 N | 8 Jul, 2014 |
| JX-FZlc-4 | *F. limnocharis* | Fuzhou City, Jiangsu | 116.31 E | 27.93 N | 8 Jul, 2014 |
| JX-FZlc-5 | *F. limnocharis* | Fuzhou City, Jiangsu | 116.31 E | 27.93 N | 8 Jul, 2014 |
| JX-JA-1 | *P. nigromaculatus* | Jian City, Jiangsu | 115.14 E | 27.23 N | 4 Aug, 2016 |
| JX-JA-2 | *P. nigromaculatus* | Jian City, Jiangsu | 115.14 E | 27.23 N | 4 Aug, 2016 |
| JX-JA-3 | *P. nigromaculatus* | Jian City, Jiangsu | 115.14 E | 27.23 N | 4 Aug, 2016 |
| JX-JJ-2 | *P. nigromaculatus* | Jiujiang City, Jiangsu | 116.05 E | 29.45 N | 15 Jul, 2014 |
| JX-JJ-3 | *P. nigromaculatus* | Jiujiang City, Jiangsu | 116.05 E | 29.45 N | 15 Jul, 2014 |
| JX-JJ-4 | *P. nigromaculatus* | Jiujiang City, Jiangsu | 116.05 E | 29.45 N | 15 Jul, 2014 |
| JX-JJ-5 | *F. limnocharis* | Jiujiang City, Jiangsu | 116.05 E | 29.45 N | 15 Jul, 2014 |
| JX-JJ-6 | *F. limnocharis* | Jiujiang City, Jiangsu | 116.05 E | 29.45 N | 15 Jul, 2014 |
| JX-YC-2 | *P. nigromaculatus* | Yichun City, Jiangsu | 114.80 E | 28.39 N | 26 Aug, 2016 |
| JX-YC-3 | *P. nigromaculatus* | Yichun City, Jiangsu | 114.80 E | 28.39 N | 26 Aug, 2016 |
| JX-YC-4 | *P. nigromaculatus* | Yichun City, Jiangsu | 114.80 E | 28.39 N | 26 Aug, 2016 |
| SC-DZ-1 | *P. nigromaculatus* | Dazhou City, Sichuan | 107.45 E | 31.21 N | 18 Jul, 2015 |
| SC-DZ-2 | *P. nigromaculatus* | Dazhou City, Sichuan | 107.45 E | 31.21 N | 18 Jul, 2015 |
| SC-GA-1 | *P. nigromaculatus* | Guangan City, Sichuan | 106.93 E | 30.33 N | 26 Jul, 2014 |
| SC-GA-2 | *P. nigromaculatus* | Guangan City, Sichuan | 106.93 E | 30.33 N | 26 Jul, 2014 |
| SC-GA-3 | *P. nigromaculatus* | Guangan City, Sichuan | 106.93 E | 30.33 N | 26 Jul, 2014 |
| SC-GA-4 | *P. nigromaculatus* | Guangan City, Sichuan | 106.93 E | 30.33 N | 26 Jul, 2014 |
| SC-GA-5 | *F. limnocharis* | Guangan City, Sichuan | 106.93 E | 30.33 N | 26 Jul, 2014 |
| SC-GA-6 | *O. margaretae* | Guangan City, Sichuan | 106.93 E | 30.33 N | 26 Jul, 2014 |
| SC-GA-7 | *O. margaretae* | Guangan City, Sichuan | 106.93 E | 30.33 N | 26 Jul, 2014 |
| SC-GA-8 | *F. limnocharis* | Guangan City, Sichuan | 106.93 E | 30.33 N | 26 Jul, 2014 |
| SC-LS-1 | *P. nigromaculatus* | Leshan City, Sichuan | 103.73 E | 29.57 N | 15 Jul, 2015 |
| SC-LS-2 | *P. nigromaculatus* | Leshan City, Sichuan | 103.73 E | 29.57 N | 15 Jul, 2015 |
| SC-LS-3 | *P. nigromaculatus* | Leshan City, Sichuan | 103.73 E | 29.57 N | 15 Jul, 2015 |
| SC-LS-4 | *B. guentheri* | Leshan City, Sichuan | 103.73 E | 29.57 N | 15 Jul, 2015 |
| SC-LS-5 | *O. margaretae* | Leshan City, Sichuan | 103.73 E | 29.57 N | 15 Jul, 2015 |
| SC-LSZ-1 | *P. nigromaculatus* | Liangshanzhou City, Sichuan | 102.26 E | 27.88 N | 12 Jul, 2015 |
| SC-LSZ-2 | *P. nigromaculatus* | Liangshanzhou City, Sichuan | 102.26 E | 27.88 N | 12 Jul, 2015 |
| SC-LSZ-3 | *P. nigromaculatus* | Liangshanzhou City, Sichuan | 102.26 E | 27.88 N | 12 Jul, 2015 |
| SC-LSZ-4 | *O. margaretae* | Liangshanzhou City, Sichuan | 102.26 E | 27.88 N | 12 Jul, 2015 |
| SC-LSZ-5 | *O. margaretae* | Liangshanzhou City, Sichuan | 102.26 E | 27.88 N | 12 Jul, 2015 |
| SC-LSZ-6 | *B. guentheri* | Liangshanzhou City, Sichuan | 102.26 E | 27.88 N | 12 Jul, 2015 |
| SC-LZ-1 | *P. nigromaculatus* | Luzhou City, Sichuan | 105.83 E | 28.82 N | 13 Jul, 2014 |
| SC-LZ-10 | *F. limnocharis* | Luzhou City, Sichuan | 105.83 E | 28.82 N | 13 Jul, 2014 |
| SC-LZ-2 | *P. nigromaculatus* | Luzhou City, Sichuan | 105.83 E | 28.82 N | 13 Jul, 2014 |
| SC-LZ-3 | *P. nigromaculatus* | Luzhou City, Sichuan | 105.83 E | 28.82 N | 13 Jul, 2014 |
| SC-LZ-4 | *P. nigromaculatus* | Luzhou City, Sichuan | 105.83 E | 28.82 N | 13 Jul, 2014 |
| SC-LZ-5 | *P. nigromaculatus* | Luzhou City, Sichuan | 105.83 E | 28.82 N | 13 Jul, 2014 |
| SC-LZ-6 | *O. margaretae* | Luzhou City, Sichuan | 105.83 E | 28.82 N | 13 Jul, 2014 |
| SC-LZ-7 | *B. guentheri* | Luzhou City, Sichuan | 105.83 E | 28.82 N | 13 Jul, 2014 |
| SC-LZ-8 | *F. limnocharis* | Luzhou City, Sichuan | 105.83 E | 28.82 N | 13 Jul, 2014 |
| SC-LZ-9 | *F. limnocharis* | Luzhou City, Sichuan | 105.83 E | 28.82 N | 13 Jul, 2014 |
| SC-NC-1 | *P. nigromaculatus* | Nanchong City, Sichuan | 106.08 E | 30.78 N | 5 Jul, 2014 |
| SC-NC-2 | *P. nigromaculatus* | Nanchong City, Sichuan | 106.08 E | 30.78 N | 5 Jul, 2014 |
| SC-NC-3 | *P. nigromaculatus* | Nanchong City, Sichuan | 106.08 E | 30.78 N | 5 Jul, 2014 |
| SC-NC-4 | *P. nigromaculatus* | Nanchong City, Sichuan | 106.08 E | 30.78 N | 5 Jul, 2014 |
| SC-NC-5 | *F. limnocharis* | Nanchong City, Sichuan | 106.08 E | 30.78 N | 5 Jul, 2014 |
| SC-NC-6 | *O. margaretae* | Nanchong City, Sichuan | 106.08 E | 30.78 N | 5 Jul, 2014 |
| SC-NC-7 | *B. guentheri* | Nanchong City, Sichuan | 106.08 E | 30.78 N | 5 Jul, 2014 |
| SC-NC-8 | *B. guentheri* | Nanchong City, Sichuan | 106.08 E | 30.78 N | 5 Jul, 2014 |
| SC-NCys-1 | *P. nigromaculatus* | Nanchong City, Sichuan | 106.57 E | 31.08 N | 5 Jul, 2014 |
| SC-NCys-2 | *P. nigromaculatus* | Nanchong City, Sichuan | 106.57 E | 31.08 N | 5 Jul, 2014 |
| SC-NCys-3 | *P. nigromaculatus* | Nanchong City, Sichuan | 106.57 E | 31.08 N | 5 Jul, 2014 |
| SC-NCys-4 | *B. guentheri* | Nanchong City, Sichuan | 106.57 E | 31.08 N | 5 Jul, 2014 |
| SC-NCys-5 | *B. guentheri* | Nanchong City, Sichuan | 106.57 E | 31.08 N | 5 Jul, 2014 |
| SC-ZG-1 | *P. nigromaculatus* | Zigong City, Sichuan | 104.81 E | 29.34 N | 31 Jul, 2015 |
| SC-ZG-2 | *P. nigromaculatus* | Zigong City, Sichuan | 104.81 E | 29.34 N | 31 Jul, 2015 |
| SC-ZG-3 | *O. margaretae* | Zigong City, Sichuan | 104.81 E | 29.34 N | 31 Jul, 2015 |
| SH-NH-1 | *P. nigromaculatus* | Nanhui City, Shanghai | 121.85 E | 30.86 N | 21 Jul, 2016 |
| SH-NH-2 | *P. nigromaculatus* | Nanhui City, Shanghai | 121.85 E | 30.86 N | 21 Jul, 2016 |
| SH-NH-3 | *P. nigromaculatus* | Nanhui City, Shanghai | 121.85 E | 30.86 N | 21 Jul, 2016 |
| SH-NH-4 | *S. latouchii* | Nanhui City, Shanghai | 121.85 E | 30.86 N | 21 Jul, 2016 |
| YN-BS-1 | *P. nigromaculatus* | Baoshan City, Yunnan | 98.50 E | 25.03 N | 25 Jul, 2014 |
| YN-BS-2 | *P. nigromaculatus* | Baoshan City, Yunnan | 98.50 E | 25.03 N | 25 Jul, 2014 |
| YN-BS-3 | *B. guentheri* | Baoshan City, Yunnan | 98.50 E | 25.03 N | 25 Jul, 2014 |
| YN-BS-4 | *B. guentheri* | Baoshan City, Yunnan | 98.50 E | 25.03 N | 25 Jul, 2014 |
| YN-HH-1 | *P. nigromaculatus* | Honghe City, Yunnan | 103.36 E | 23.40 N | 17 Jul, 2016 |
| YN-HH-2 | *P. nigromaculatus* | Honghe City, Yunnan | 103.36 E | 23.40 N | 17 Jul, 2016 |
| YN-KM-1 | *P. nigromaculatus* | Kunming City, Yunnan | 102.72 E | 25.05 N | 23 Jul, 2014 |
| YN-KM-2 | *P. nigromaculatus* | Kunming City, Yunnan | 102.72 E | 25.05 N | 23 Jul, 2014 |
| YN-KM-3 | *P. nigromaculatus* | Kunming City, Yunnan | 102.72 E | 25.05 N | 23 Jul, 2014 |
| YN-KM-4 | *P. nigromaculatus* | Kunming City, Yunnan | 102.72 E | 25.05 N | 23 Jul, 2014 |
| YN-KM-5 | *B. guentheri* | Kunming City, Yunnan | 102.72 E | 25.05 N | 23 Jul, 2014 |
| YN-KM-6 | *B. guentheri* | Kunming City, Yunnan | 102.72 E | 25.05 N | 23 Jul, 2014 |
| YN-WS-1 | *P. nigromaculatus* | Wenshan City, Yunnan | 104.34 E | 23.61 N | 5 Aug, 2016 |
| YN-WS-2 | *B. guentheri* | Wenshan City, Yunnan | 104.34 E | 23.61 N | 5 Aug, 2016 |
| ZJ-JX-1 | *P. nigromaculatus* | Jiaxing City, Zhejiang | 121.02 E | 30.7 N | 12 Jul, 2014 |
| ZJ-JX-2 | *P. nigromaculatus* | Jiaxing City, Zhejiang | 121.02 E | 30.7 N | 12 Jul, 2014 |
| ZJ-JX-3 | *F. limnocharis* | Jiaxing City, Zhejiang | 121.02 E | 30.7 N | 12 Jul, 2014 |
| ZJ-NBbl-1 | *P. nigromaculatus* | Ningbo City, Zhejiang | 121.85 E | 29.93 N | 9 Aug, 2014 |
| ZJ-NBbl-2 | *F. limnocharis* | Jiaxing City, Zhejiang | 121.85 E | 29.93 N | 9 Aug, 2014 |
| ZJ-NBbl-3 | *S. latouchii* | Jiaxing City, Zhejiang | 121.85 E | 29.93 N | 9 Aug, 2014 |
| ZJ-NBcx-1 | *P. nigromaculatus* | Jiaxing City, Zhejiang | 121.23 E | 30.17 N | 4 Aug, 2014 |
| ZJ-NBcx-2 | *P. nigromaculatus* | Jiaxing City, Zhejiang | 121.23 E | 30.17 N | 4 Aug, 2014 |
| ZJ-NBcx-3 | *P. nigromaculatus* | Jiaxing City, Zhejiang | 121.23 E | 30.17 N | 4 Aug, 2014 |
| ZJ-NBcx-4 | *P. nigromaculatus* | Jiaxing City, Zhejiang | 121.23 E | 30.17 N | 4 Aug, 2014 |
| ZJ-NBcx-5 | *S. latouchii* | Jiaxing City, Zhejiang | 121.23 E | 30.17 N | 4 Aug, 2014 |
| ZJ-NBcx-6 | *S. latouchii* | Jiaxing City, Zhejiang | 121.23 E | 30.17 N | 4 Aug, 2014 |
| ZJ-NBcx-7 | *F. limnocharis* | Jiaxing City, Zhejiang | 121.23 E | 30.17 N | 4 Aug, 2014 |
| ZJ-NBcx-8 | *F. limnocharis* | Jiaxing City, Zhejiang | 121.23 E | 30.17 N | 4 Aug, 2014 |
| ZJ-NByy-1 | *P. nigromaculatus* | Jiaxing City, Zhejiang | 121.15 E | 30.03 N | 13 Jul, 2014 |
| ZJ-NByy-2 | *P. nigromaculatus* | Jiaxing City, Zhejiang | 121.15 E | 30.03 N | 13 Jul, 2014 |
| ZJ-NByy-3 | *S. latouchii* | Jiaxing City, Zhejiang | 121.15 E | 30.03 N | 13 Jul, 2014 |
| ZJ-SX-2 | *P. nigromaculatus* | Shaoxing City, Zhejiang | 120.47 E | 30.08 N | 21 Jul, 2014 |
| ZJ-SX-3 | *P. nigromaculatus* | Shaoxing City, Zhejiang | 120.47 E | 30.08 N | 21 Jul, 2014 |
| ZJ-SX-4 | *P. nigromaculatus* | Shaoxing City, Zhejiang | 120.47 E | 30.08 N | 21 Jul, 2014 |
| ZJ-SX-5 | *S. latouchii* | Shaoxing City, Zhejiang | 120.47 E | 30.08 N | 21 Jul, 2014 |
| ZJ-SX-6 | *S. latouchii* | Shaoxing City, Zhejiang | 120.47 E | 30.08 N | 21 Jul, 2014 |
| ZJ-ZJ-1 | *P. nigromaculatus* | Zhuji City, Zhejiang | 120.38 E | 29.58 N | 20 Jul, 2016 |
| ZJ-ZJ-2 | *P. nigromaculatus* | Zhuji City, Zhejiang | 120.38 E | 29.58 N | 20 Jul, 2016 |
| ZJ-ZJ-3 | *P. nigromaculatus* | Zhuji City, Zhejiang | 120.38 E | 29.58 N | 20 Jul, 2016 |
| ZJ-ZJ-4 | *P. nigromaculatus* | Zhuji City, Zhejiang | 120.38 E | 29.58 N | 20 Jul, 2016 |
| ZJ-ZJ-5 | *S. latouchii* | Zhuji City, Zhejiang | 120.38 E | 29.58 N | 20 Jul, 2016 |
| ZJ-ZJ-6 | *S. latouchii* | Zhuji City, Zhejiang | 120.38 E | 29.58 N | 20 Jul, 2016 |
| ZJ-ZJ-7 | *F. limnocharis* | Zhuji City, Zhejiang | 120.38 E | 29.58 N | 20 Jul, 2016 |
| ZJ-ZJ-8 | *F. limnocharis* | Zhuji City, Zhejiang | 120.38 E | 29.58 N | 20 Jul, 2016 |

**Table S2.** Primers of cytochrome c oxidase subunit 1 (*cox1*) gene.

| Gene | Name | Sequence (5′–3′) | References |
| --- | --- | --- | --- |
| *cox1* | cox1F | TAGACTAAGTGTTTTCAAAACACTA | Yanagida et al. (2010) |
|  | cox1R | ATAGCATGATGCAAAAGG |  |

**Table S3.** SSR mining in *S. mansoni*.

| SSR mining statistics | |
| --- | --- |
| Total number of sequences examined | 109,272 |
| Total number of identified SSRs | 4,280 |
| Distribution of SSRs in different repeat types |  |
| di-nucleotide | 1,024 |
| tri-nucleotide | 2,720 |
| tetra-nucleotide | 101 |

**Table S4.** Characteristics of a core set of 23 SSR markers.

| **Target** | **start** | **end** | **ref_motif** | ***Na*** | ***Ho*** | ***He*** | **PIC** | **Saturation** |
| --- | --- | --- | --- | --- | --- | --- | --- | --- |
| T_04 | 91 | 108 | (ACT)6 | 17 | 0.48 | 0.91 | 0.90 | 16.90 |
| T_59 | 90 | 101 | (ACG)4 | 7 | 0.67 | 0.78 | 0.74 | 36.57 |
| T_05 | 67 | 84 | (GCT)6 | 4 | 0.40 | 0.46 | 0.39 | 50.69 |
| T_27 | 54 | 65 | (CGA)4 | 4 | 0.66 | 0.52 | 0.48 | 60.66 |
| T_02 | 86 | 97 | (CAC)4 | 8 | 0.89 | 0.77 | 0.73 | 67.87 |
| T_24 | 80 | 91 | (ACC)4 | 5 | 0.87 | 0.69 | 0.64 | 71.75 |
| T_32 | 38 | 55 | (GAG)6 | 5 | 0.20 | 0.53 | 0.49 | 74.52 |
| T_41 | 86 | 103 | (GCA)6 | 9 | 0.85 | 0.73 | 0.69 | 77.84 |
| T_30 | 86 | 100 | (GCA)5 | 4 | 0.45 | 0.44 | 0.40 | 80.06 |
| T_35 | 95 | 109 | (CAG)5 | 6 | 0.87 | 0.63 | 0.57 | 81.99 |
| T_40 | 58 | 72 | (CGA)5 | 6 | 0.42 | 0.64 | 0.57 | 83.66 |
| T_36 | 74 | 88 | (GAG)5 | 6 | 0.96 | 0.69 | 0.63 | 84.76 |
| T_63 | 87 | 107 | (CTC)7 | 5 | 0.85 | 0.61 | 0.54 | 85.87 |
| T_45 | 54 | 65 | (AGAC)3 | 3 | 0.29 | 0.50 | 0.39 | 86.43 |
| T_64 | 106 | 117 | (GCA)4 | 4 | 0.33 | 0.36 | 0.33 | 87.26 |
| T_39 | 30 | 41 | (AGC)4 | 8 | 0.70 | 0.59 | 0.54 | 88.37 |
| T_23 | 49 | 60 | (GGA)4 | 4 | 0.50 | 0.62 | 0.54 | 88.92 |
| T_61 | 84 | 95 | (GAT)4 | 3 | 0.89 | 0.55 | 0.45 | 89.47 |
| T_08 | 30 | 41 | (GCAC)3 | 3 | 0.60 | 0.63 | 0.55 | 90.03 |
| T_57 | 53 | 70 | (ACG)6 | 5 | 0.46 | 0.57 | 0.52 | 90.30 |
| T_01 | 76 | 87 | (GCT)4 | 5 | 0.75 | 0.60 | 0.52 | 90.58 |
| T_60 | 75 | 86 | (CTT)4 | 4 | 0.32 | 0.31 | 0.29 | 90.86 |
| T_62 | 83 | 97 | (ATG)5 | 5 | 0.55 | 0.52 | 0.43 | 91.14 |

**Table S5.** Analysis of molecular variance (AMOVA) of the populations of *S. mansoni* based on a core set of 23 SSRs.

| Source of variation | d.f. | Sum of squares | Variance components | Percentage of variation |
| --- | --- | --- | --- | --- |
| Among groups | 2 | 77.193 | 0.13265 | 1.98358 |
| Among populations within groups | 13 | 213.034 | 0.23831 | 3.56359 |
| Within populations | 346 | 4343.411 | 6.31645 | 94.45283 |
| Total | 361 | 4633.638 | 6.68741 | 100 |

d.f.=degrees of freedom.

**Table S6.** Core samples of *S. mansoni* identified in each geographical population of China.

| Population | Core sample |
| --- | --- |
| AH | AH-HF-2, AH-HF-4, AH-LA-3, AH-LA-5 |
| CQ | CQ-FL-1, CQ-FL-2, CQ-LP-3, CQ-LP-4 |
| FJ | FJ-NP-1 |
| GD | GD-DG-1, GD-GZ-2 |
| GX | GX-GL-3, GX-GL-4, GX-NN-5, GX-WZ-6, GX-WZ-11 |
| GZ | GZ-AS-1, GZ-GY-3, GZ-GY-5, GZ-KL-8 |
| HB | HB-HG-2, HB-HG-3 |
| HeN | HeN-LH-4 |
| HN | HN-WZS-8 |
| HuN | HuN-CS-1, HuN-HH-2, HuN-HH-3, HuN-SY-1, HuN-XX-2, HuN-XXfh-1, HuN-XXfh-6, HuN-YY-1, HuN-CD-3, HuN-CS-4, HuN-CS-8, HuN-CS-12, HuN-CZ-1, HuN-LD-1, HuN-LD-2, HuN-SY-5, HuN-YiY-2, HuN-YZ-2, HuN-ZJJ-5, HuN-ZJJ-6 |
| JS | JS-SZ-1, JS-SZ-4, JS-SZ-6, JS-ZJ-5 |
| JX | JX-FZcg-2, JX-FZcg-5, JX-FZlc-3, JX-JA-2, JX-YC-3 |
| SC | SC-DZ-1, SC-GA-3, SC-LS-3, SC-LS-5, SC-LZ-2, SC-LZ-7, SC-NC-1, SC-NC-7, SC-NCys-5 |
| SH | SH-NH-3 |
| YN | YN-BS-1, YN-BS-2, YN-BS-3, YN-BS-4, YN-HH-2, YN-WS-1 |
| ZJ | ZJ-NBbl-3, ZJ-NBcx-1, ZJ-NByy-3 |

**Figure S1.** Estimates of pairwise *F*_st_ between *S. mansoni*
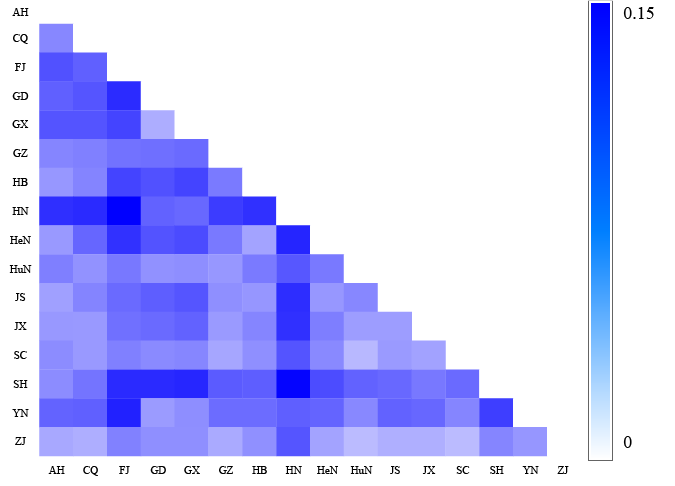
 populations.


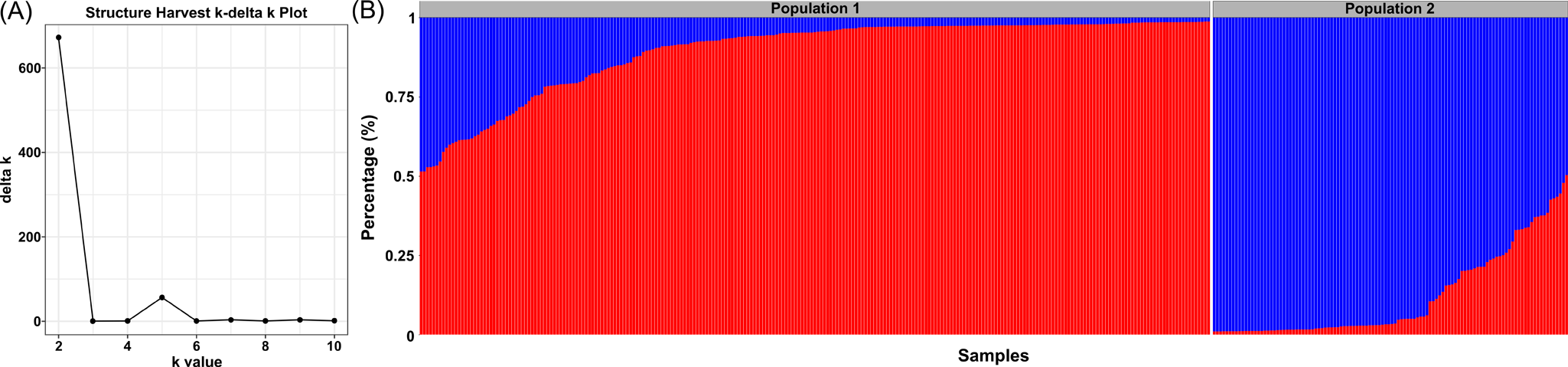


**Figure S2.** Estimated genetic structure of *S. mansoni* in China as inferred by the STRUCTURE software on the basis of the data on a core set of 23 SSRs. (A) Plot of the delta *K* values generated by the STRUCTURE. (B) STRUCTURE plots of 362 individuals grouped by the Q-matrix (estimated membership coefficient for each sample) at *K* = 2. Each isolate is represented by a vertical line, partitioned into the colored segments representing the tapeworm estimated membership fractions in *K*. The same color indicates that the isolates belong to the same group. Different colors for the same isolate indicate the percentage of the genotype shared with each group.


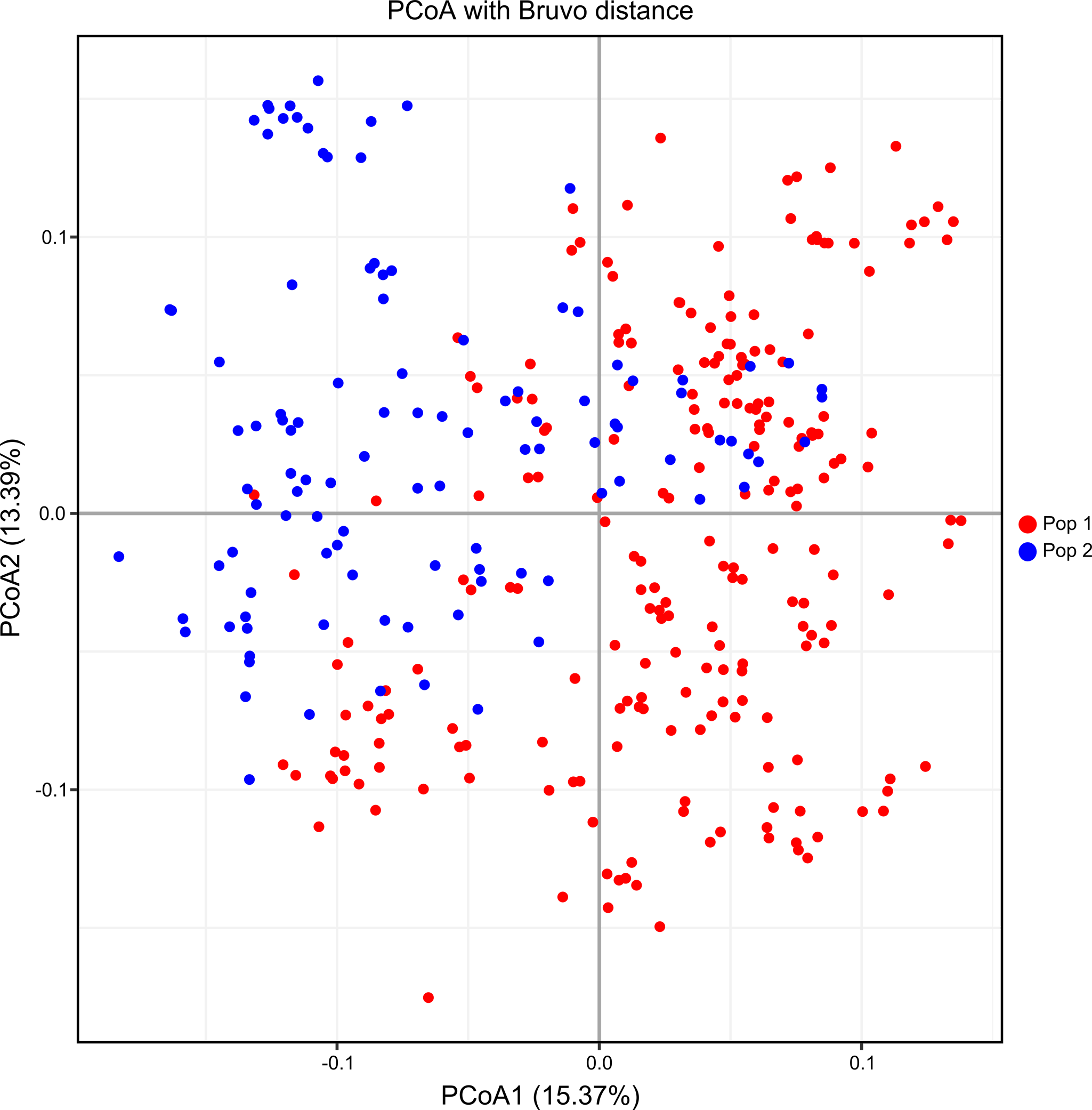


**Figure S3.** Principal coordinate analysis (PCoA) describing the relationships of 362 *S. mansoni* isolates on the basis of the data on a core set of 23 SSRs. Pop1 and Pop2 are labeled in red and blue, respectively.
